# A nowhere-to-hide mechanism ensures complete piRNA-directed DNA methylation

**DOI:** 10.1038/s41586-025-09940-w

**Published:** 2026-01-14

**Authors:** Tamoghna Chowdhury, Shelagh Boyle, Ansgar Zoch, Xinyu Xiang, Madeleine Dias Mirandela, Hanna Fieler, Christos Spanos, Juan Zou, David Kelly, Wendy A. Bickmore, Atlanta G. Cook, Dónal O’Carroll

**Affiliations:** 1 Institute for Regeneration and Repair, Institute for Stem Cell Research, University of Edinburgh, 5 Little France Drive, Edinburgh, EH16 4UU, UK; 2 Centre for Cell Biology, University of Edinburgh, Michael Swann Building, Max Born Crescent, Edinburgh, EH9 3BF, UK; 3 MRC Human Genetics Unit, Institute of Genetics and Cancer, University of Edinburgh, Crewe Road South, Edinburgh EH4 2XU, Edinburgh, UK; 4 Zhejiang University-University of Edinburgh Institute (ZJU-UoE Institute), Zhejiang University School of Medicine, International Campus, Zhejiang University, 718 East Haizhou Road, Haining 314400, China

**Keywords:** Germline, piRNA, PIWI proteins, transposons, SPOCD1, TPR

## Abstract

The mouse PIWI-interacting RNA (piRNA) pathway provides sustained anti-transposon immunity to the developing male germline by directing transposon DNA methylation ^1–3^. The first step in this process is the recruitment of SPOCD1 to young LINE1 loci ^4^. Thereafter, piRNA-mediated tethering of the PIWI protein MIWI2 (PIWIL4) to the nascent transposon transcript recruits the DNA methylation machinery ^5,6^. The piRNA pathway needs to methylate all active transposon copies but how this is achieved remains unknown. Here, we show that nuclear piRNA and *de novo* methylation factors are all euchromatic, exposing constitutive heterochromatin as a genomic blind spot for the piRNA pathway. We discover a ‘nowhere-to-hide’ mechanism that enables piRNA pathway-mediated LINE1 surveillance of the entire genome. We find that SPOCD1 directly interacts with the nuclear pore component TPR, which forms heterochromatin exclusion zones adjacent to nuclear pores ^7^. In foetal gonocytes undergoing piRNA-directed DNA methylation, TPR is found both at the nuclear periphery and throughout the nucleoplasm. We find that the SPOCD1-TPR interaction is required for complete non-stochastic piRNA-directed LINE1 methylation. The loss of the SPOCD1-TPR interaction results in a fraction of SPOCD1 and other chromatin-bound piRNA factors relocalising to constitutive heterochromatin where they are no longer accessible to MIWI2 and the *de novo* methylation machinery. In summary, the piRNA pathway has co-opted TPR to guarantee LINE1s are accessible to the piRNA and *de novo* methylation machineries.

The germline is an ancient cell lineage responsible for the continuity of animal life. Active transposons threaten the continuity of the germline through transposition-associated DNA damage and mutation. Promoter DNA methylation is a fundamental mechanism that mediates transposon repression ^8^. However, during embryonic development the mammalian germline undergoes reprogramming and the erasure of genomic DNA methylation ^9–12^. Reprogramming unleashes transposons and left unchecked they will annihilate the germline ^1,2,13^. The piRNA pathway provides anti-transposon immunity to the germline during this period of extreme vulnerability ^14^. piRNAs are PIWI protein-bound small RNAs that through base complementarity act as guides to recruit PIWI proteins to cellular RNAs ^14^. In the cytoplasm, piRNAs guide PIWI-mediated destruction of transposon transcripts to neutralise the acute threat posed by transposon derepression ^5,15,16^. In the nucleus, the piRNA pathway directs transposon silencing through promoter *de novo* methylation ^1–3^. Through base complementarity, piRNAs tether the PIWI protein MIWI2 to nascent transposon transcripts ^5,6^, which ultimately results in the recruitment of the *de novo* methylation machinery - the methyltransferase DNMT3C and its cofactor DNMT3L ^13,17–20^. SPOCD1 is a scaffold protein and with C19ORF84 connects MIWI2 to the DNA methylation machinery^19,20^. The precision of the piRNA pathway is essential for the genetic and epigenetic integrity of the germline: the system must ensure that all active transposon copies are methylated while simultaneously avoiding off-target methylation. A two-step process prevents off-targeting of active Long Interspersed Nuclear Element-1 (LINE1) transposon copies ^4^. The chromatin modification H3K4me3-K9me3 marks active LINE1s in developing foetal gonocytes that recruits SPOCD1 through the reader protein SPIN1, an essential licencing step that precedes MIWI2 engagement ^4^. However, how the piRNA pathway ensures that all active transposons are methylated remains unknown.

The clue to how the piRNA pathway ensures complete LINE1 DNA methylation came from nuclear localisation observations. Embryonic day 16.5 (E16.5) foetal germ cells that are undergoing *de novo* genome methylation have a different heterochromatin configuration compared to most somatic cells^21^. They lack chromo centres or punctate heterochromatic foci (Extended Data Fig. 1a) ^21^. Instead, heterochromatin, as defined by H3K9me3 or HP1 proteins, is found at the nuclear periphery or adjacent to nucleoli (Extended Data Fig. 1a) ^21^. Using the HA epitope-tagged *Spocd1*^*HA*^ allele ^19^, we found that SPOCD1 is excluded from constitutive heterochromatin in foetal germ cells (Fig. 1a and Extended Data Fig. 1b) and largely appears to colocalise with H3K4me3 (Fig. 1a and Extended Data Fig. 1b), a marker of active transcription and euchromatin ^22^. To understand if heterochromatin exclusion is a general property of piRNA pathway factors, we employed the previously published endogenously HA tagged *Miwi2*^*HA*^, *Dnmt3l*^*HA*^ and *Dnmt3c*^*HA*^ alleles ^20,23^ that allow the use of the same antibody to detect all factors with the same sensitivity. MIWI2 and the *de novo* methylation machinery components DNMT3L and DNMT3C are also depleted at constitutive heterochromatin but colocalize with euchromatin (Fig. 1b-d and Extended Data Fig. c-e). Furthermore, C19ORF84 and SPIN1 show the same euchromatic nuclear localization (Fig. 1e-f and Extended Data Fig. 1f-g). To understand if the euchromatic association of piRNA factors occurs throughout this process, we examined the colocalization of MIWI2, DNMT3L and SPOCD1 with H3K4me3 across multiple developmental time points. We found that the colocalization of these piRNA factors with H3K4me3 was observed upon initiation of their expression and throughout the period of piRNA-directed DNA methylation (Extended Data Fig. 2a-c). In summary, the piRNA and *de novo* methylation machineries predominantly operate in euchromatin.

The exclusion of piRNA factors from heterochromatic regions of the nucleus exposes a vulnerability in the piRNA pathway and to the germline: should an unmethylated transposon locus through proximity get incorporated into a constitutive heterochromatic environment it would be inaccessible to the pathway and could escape DNA methylation. Given that transposition is uncontrolled, it raises the question of how the piRNA pathway eliminates safe harbours or immune-privileged sites for active transposons. We therefore hypothesised that an active mechanism should be in place to eradicate such genomic blind spots. We focused on SPOCD1 because, through SPIN1, it directly binds active LINE1 loci and functions as a molecular scaffold ^4,19,20^. We therefore interrogated previously published SPOCD1 IP-MS datasets from E16.5 foetal testes ^19^ for factors with a role in heterochromatin exclusion and found TPR (Fig. 2a). TPR is a ubiquitously expressed protein that forms parts of the nuclear basket of the nuclear pore complex ^24–27^. Intriguingly, TPR is required for limiting heterochromatin distribution by forming heterochromatin exclusion zones in the vicinity of the nuclear pore ^7^. MIWI2 and C19ORF84 also associate with TPR at E16.5 (Extended Data Fig. 3a-b) ^19,20^. No other nuclear pore components were identified in SPOCD1 or C19ORF84 IP-MS datasets ^19,20^ whereas NUP98 was identified in one of the four published MIWI2 datasets^6,19^. IP-MS of TPR from E16.5 foetal testis revealed its association with SPOCD1 but not MIWI2 (Fig. 2b), indicating that SPOCD1 and TPR could directly interact. Ectopically expressed SPOCD1 associates with endogenous TPR in mouse embryonic stem cells and this association is dependent upon the TFIISM domain of SPOCD1 but not its SPOC domain (Fig. 2c). TPR is a large 267 kDa protein comprised of an N-terminal coiled-coil domain and an acidic, disordered C-terminal domain ^28^. To understand which portion of TPR associates with the SPOCD1-TFIISM domain, we expressed previously defined ^28^ N-terminal (TPR-N), an internal (TPR-M) and C-terminal (TPR-C) fragments of TPR in human embryonic kidney (HEK) 293 cells. The recombinant SPOCD1 TFIISM domain pulled down the TPR-M fragment from HEK cell lysate (Fig. 2d). Cross-linking mass spectrometry (CL-MS) revealed that amino acids 775 to 877 of TPR interact with the TFIISM domain of SPOCD1 (Fig. 2e and Supplementary Table 3). Strikingly, TPR is highly expressed in E16.5 foetal gonocytes during piRNA-directed DNA methylation (Fig. 2f and Extended Data Fig. 3c-d) and the level of TPR expression is far higher in the germ cells than in the neighbouring somatic cells (Extended Data Fig. 3c-d). Furthermore, TPR is abundantly expressed in foetal gonocytes immediately prior to and during the process of piRNA-directed DNA methylation (Extended Data Fig. 3e-f). TPR forms a characteristic ring around the nucleus, but it is also abundantly found within the nucleoplasm, which is evident at higher exposures (Fig. 2f). Indeed, TPR and SPOCD1 colocalise to a degree in the nucleoplasm and nuclear periphery (Fig. 2f). In summary, TPR directly interacts with SPOCD1 and is abundantly expressed in foetal gonocytes undergoing piRNA-directed DNA methylation.

Given that TPR directly interacts with SPOCD1 and has the intrinsic ability to form heterochromatin exclusion zones ^7^, we wanted to understand if TPR participates in piRNA-directed transposon methylation. *Tpr* is an essential gene (https://www.informatics.jax.org/marker/phenotypes/MGI:1922066) being part of the nuclear pore basket and required for the nuclear export of intronless and histone mRNAs ^25,29,30^. As SPOCD1’s function is restricted to piRNA-directed DNA methylation and not required for mouse viability ^19^, we therefore sought to define a SPOCD1 separation-of-function mutation that can no longer interact with TPR. Analysis of SPOCD1’s TFIISM domain revealed highly conserved lysine residues that are surface exposed in AlphaFold models and crosslink with TPR in the CL-MS experiment (Fig. 3a and Extended Data Fig. 4a). Mutation of SPOCD1 lysine 464 to alanine (SPOCD1-K464A) abrogated TPR interaction in recombinant protein pull down assays (Fig. 3b). Based on the CL-MS data we identified two negatively charged TPR residues (E830 and E832) that were candidates to mediate an electrostatic interaction with the positively charged patch on the SPOCD1-TFIISM domain. Mutation of TPR E830 and E832 to alanine (TPR-E830A/E832A) abrogates the interaction with SPOCD1 (Extended Data Fig. 4b). To understand if the SPOCD1-TPR interaction is required for piRNA-directed transposon methylation, we generated a mouse *Spocd1*^*K464A*^ allele (Extended Data Fig. 5a-b). *Spocd1*^*K464A/K464A*^ (abbreviated to *Spocd1*^*K464A*^) male mice expressed normal levels of SPOCD1, TPR and MIWI2 but were sterile (Fig. 3c-d). Importantly, SPOCD1 IP-MS experiments revealed that the SPOCD1-TPR interaction is lost in *Spocd1*^*K464A*^ foetal gonocytes (Extended Data Fig. 5c). *Spocd1*^*K464A*^ mice have atrophic testes and lack sperm in the epididymis (Fig. 3e). Histological analysis revealed a variety of spermatogenic phenotypes (Fig. 3f) ranging from seminiferous tubules lacking germ cells, also known as Sertoli-only, to arrested spermatogenesis. Most tubules showed a meiotic arrest with a tiny fraction of tubules arresting in spermiogenesis (Fig. 3f). DNA damage and apoptosis were broadly detected in *Spocd1*^*K464A*^ seminiferous tubules (Extended Data Fig. 5d). In summary, the SPOCD1-TPR interaction is required for male mouse fertility.

SPOCD1 is required for the DNA methylation and silencing of both young LINE1 and Intracisternal A-Particle (IAP) transposon copies ^19^. In adult *Spocd1*^*K464A*^ testes, expression of the LINE1 ORF1p but not IAP GAG expression is observed (Fig. 3g). We next analysed DNA methylation from undifferentiated spermatogonia isolated from postnatal day 14 testis, a timepoint commonly studied because it is prior to the onset of the spermatogenic phenotype and after the completion of genome methylation ^4,6,17,19,20,23,31,32^. The SPOCD1-TPR interaction is not required for the general genome methylation (Fig. 3h) as is the case for disruption of other nuclear piRNA pathway factors ^4,6,17,19,20^. However, young LINE1 families (L1Md_A, L1Md_T and L1Md_Gf) were hypomethylated in *Spocd1*^*K464A*^ spermatogonia in contrast to the near-wildtype methylation levels in the old LINE1 L1Md_F family or the IAPEz or IAPEy families in *Spocd1*^*K464A*^ spermatogonia (Fig. 3h). The piRNA pathway specifically methylates transposon promoters ^17^. Indeed, defective *de novo* promoter methylation of the young L1Md_A, L1Md_T and L1Md_Gf LINE1 families is observed in *Spocd1*^*K464A*^ spermatogonia (Fig. 3i). We next analysed hypomethylation at individual transposon loci in relation to divergence from the consensus sequence, a proxy for transposon age. This revealed that the SPOCD1-TPR interaction is required for the methylation of young LINE1 elements (Fig. 3j). In conclusion, the SPOCD1-TPR interaction is essential for piRNA-directed LINE1 methylation. We next investigated the relationship between SPOCD1 and constitutive heterochromatin in E16.5 *Spocd1*^*K464A*^ foetal gonocytes. Strikingly, in a fraction of E16.5 *Spocd1*^*K464A*^ foetal gonocytes a portion of SPOCD1 relocalises to constitutive heterochromatin to form foci (Fig. 4a and Extended Data Fig. 6a). In addition to TPR, SPOCD1 directly interacts with SPIN1 and C19ORF84 ^4,20^; both SPIN1 and C19ORF84 also localised to heterochromatic foci in *Spocd1*^*K464A*^ gonocytes (Fig. 4b-c and Extended Data Fig. 6b-c). Consistently, we also found that H3K4me3 colocalises with SPOCD1 and HP1B in the heterochromatic foci in E16.5 *Spocd1*^*K464A*^ foetal gonocytes (Extended Data Fig. 6d). However, neither MIWI2 nor the *de novo* methylation machinery components DNMT3L and DNMT3C localise at heterochromatin foci (Fig. 4d-f and Extended Data Fig. 6e-g). This implies that a fraction of LINE1 copies could get incorporated into heterochromatin during the period of *de novo* methylation. We were not able to detect individual LINE1 copies by DNA fluorescence in situ hybridisation (DNA-FISH). A full length LINE1 probe that recognises all LINE1 elements for DNA FISH coupled with immunofluorescence for SPOCD1 was used to determine whether LINE1 copies are present in the heterochromatic foci observed in *Spocd1*^*K464A*^ foetal gonocytes (Fig. 4g). LINE1 signal is found throughout the nucleus and colocalises with SPOCD1 in wild-type cells (Fig. 4g) Importantly, LINE1 DNA FISH signal is found in the heterochromatic SPOCD1 foci observed in *Spocd1*^*K464A*^ foetal gonocytes (Fig. 4g). Methylation of the imprinted *Rasgrf1* locus is also dependent upon the piRNA pathway ^33^ and we found the SPOCD1-TPR interaction is required for *Rasgrf1* locus methylation (Extended Data Fig. 7a). To examine heterochromatin association at the single locus level, we used probes against *Rasgrf1* as a piRNA-target locus and a control locus on *Mus musculus* chromosome 9 that is distant from any active LINE1s for DNA-FISH analysis. Using DAPI dense staining regions as a marker for heterochromatin, we found a statistically significant increase in the number of cells where *Rasgrf1* loci were found in heterochromatin in *Spocd1*^*K464A*^ foetal gonocytes compared to wildtype (Fig. 4h). This is not the case for the control locus (Fig. 4i). In summary, the SPOCD1-TPR interaction prevents a portion of SPOCD1 and its target loci from getting incorporated into constitutive heterochromatin where they are inaccessible to both MIWI2 and the methylation machinery. We noted that the defective DNA methylation of L1Md_A, L1Md_T and L1Md_Gf was less severe in *Spocd1*^*K464A*^ spermatogonia in comparison to *Miwi2*^*-/-*^ and *C19orf84*^*-/-*^ spermatogonia (Fig. 3i). L1Md_A behaves as an outlier in *Spocd1*^*-/-*^ spermatogonia with respect to *Miwi2*^*-/-*^ and *C19orf84*^*-/-*^ spermatogonia (Fig. 3i) but the degree of defective L1Md_T and L1Md_Gf methylation was also less in *Spocd1*^*K464A*^ cells compared to *Spocd1*^*-/-*^ cells (Fig. 3i). The weaker *de novo* methylation phenotype in *Spocd1*^*K464A*^ mice could have several underlying causes. First, all LINE1s could be methylated at a lower level so the process is inefficient rather than defective. This was not the case as there were no significant differences in median methylation levels of all LINE1 families between *Spocd1*^*K464A*^ and *Spocd1*^*-/-*^ spermatogonia (Fig. 4j). Second, there could be a deterministic defect where some defined LINE1 copies absolutely require the SPOCD1-TPR interaction for their methylation. Third, the process could stochastically misfunction where the pathway fails to methylate different LINE1 copies. To discriminate between a deterministic or stochastic function for the SPOCD1-TPR interaction, we analysed the variance between the biological replicates of genomic methylation for the respective genotypes (Fig. 4k). A low variance would support a deterministic defect, and high variance would indicate a stochastic misfunction. Strikingly, the variance in L1Md_A, L1Md_T and L1Md_Gf methylation was higher in *Spocd1*^*K464A*^ spermatogonia relative to wild type, *Miwi2*^*-/-*^ or *C19orf84*^*-/-*^ spermatogonia (Fig. 4k). The same was true for L1Md_T and L1Md_Gf methylation in *Spocd1*^*K464A*^ versus *Spocd1*^*-/-*^ spermatogonia (Fig. 4k). In conclusion, the SPOCD1-TPR interaction ensures high fidelity piRNA-directed LINE1 methylation by ensuring young LINE1s and the *Rasgrf1* locus are euchromatic and accessible to MIWI2 and the *de novo* methylation machinery (Extended Data Fig. 7b).

Here we show that nuclear piRNA pathway factors and the *de novo* methylation machinery are mostly euchromatic in foetal gonocytes. This reveals that the process of piRNA-directed DNA methylation occurs in euchromatin and exposes heterochromatin as a blind spot and vulnerability for the piRNA pathway. We discover an active ‘nowhere-to-hide’ mechanism that counteracts the existence of immune-privileged sites in the genome and ensures that active LINE1 copies and *Rasgrf1* are kept euchromatic and subject to complete non-stochastic methylation. This mechanism is critical to counteract the possible heterochromatinization of active LINE1s and enables surveillance of the entire genome. Incorporation of an active LINE1 copy into a heterochromatic environment would likely lead to its transcriptional silencing in foetal gonocytes but not its DNA methylation. This eventuality would be detrimental to the germline, as DNA methylation is required for LINE1 silencing in meiosis and germ cell survival ^34^. It is interesting to note that active IAPs that are also targets of the mouse piRNA pathway ^1–3^ are not subject to this mechanism. This highlights another mechanistic difference between piRNA directed- IAP and LINE1 methylation. IAP methylation also does not rely on SPIN1 function or piRNA amplification ^4,5^. We demonstrate that SPOCD1 directly interacts with TPR, and this interaction is at the core of this nowhere-to-hide mechanism. TPR is a component of the nuclear pore basket ^25^ and required for the formation of heterochromatin exclusion zones ^7^. This property of TPR reduces the frequency of SPOCD1, SPIN1 and C19ORF84 incorporation into heterochromatin, ensuring access of LINE1s and *Rasgrf1* to MIWI2 and the methylation machinery. We speculate that this activity of TPR in piRNA-directed DNA methylation operates throughout the nucleoplasm, as evidenced by the lack of other nuclear pore proteins associated with SPOCD1 ^19^. TPR is very highly expressed in foetal germ cells undergoing *de novo* genome methylation which could be a germ cell adaptation to support complete piRNA-directed DNA methylation throughout the nucleus. TPR is also found throughout the nucleoplasm in somatic cells ^25^, which raises the possibility of a broader utilization of TPR’s heterochromatin exclusion function beyond the nuclear periphery. In summary, the piRNA pathway through SPOCD1 has co-opted TPR’s heterochromatin exclusion function to guarantee LINE1s and *Rasgrf1* are euchromatic and accessible for piRNA-directed methylation.

## Methods

### Mouse strains and experimentation

The *Spocd1*^*HA*^, *Dnmt3c*^*HA*^, *Dnmt3l*^*HA*^, and *Miwi2*^*HA*^ mouse alleles have been described previously ^4,19,20,23,35^. The *Spocd1*^*HA*^ and *Miwi2*^*HA*^ mice were kept on a mixed B6CBAF1/Crl;C57BL/6N;Hsd:ICR (CD1) genetic background, while the *Dnmt3c*^*HA*^ and *Dnmt3l*^*HA*^ mice were kept on a mixed B6CBAF1/Crl;C57BL/6N genetic background. The *Spocd1*^*K464A*^ allele was generated by CRISPR-CAS9 gene editing as previously described ^36^. A single sgRNA (TGCGGTACTTGTTCTTGTAG) together with CAS9 mRNA and a single-stranded DNA oligo containing the K464A mutation flanked by 91bp of left and 90bp of right homology arm (TCCAACCTTCAGTCTGTTCCTAGTGCCCCCCAACCCCCCCCCCCAGCTCACAACATTTC GGGGATCTCGCAGGTTGAAGAGCAGGCTGCGaTAggcGTTCTTGTAGCGcAGGTTGGTGTC TTGCGTCAGGTGGAAGAGGGCCTCCTCAATGCCCTCTGCGATGGCCTCTACCTCATCTTC CCTCAACGCCAGGTCGGGAA) was injected into the cytoplasm of fertilised 1-cell zygotes (B6CBAF1/Crl). F_0_ offspring were screened by PCR and the *Spocd1*^*K464A*^ allele was confirmed by Sanger sequencing. The allele was established from one founder animal and back-crossed multiple times to a C57BL6/6N genetic background. Thus, the *Spocd1*^*K464A*^ mice were on a mixed B6CBAF1/Crl;C57BL/6N genetic background. Mice were genotyped using a 4 primer PCR (F – TGAGCTCTCCCTAGGTAAATGG, R – TGAGCCACTTTGAGAAACAGGT, WT-F – ACCTCCGCTACAAGAACAAG, K464A-R – AAGAGCAGGCTGCGATAG).

Male fertility was assessed by mating studs to Hsd:ICR (CD1) wild-type females and counting the number of pups born for each plugged female. For each experiment, animal tissue samples were collected from one or more litters and allocated to groups according to genotype. No further randomization or blinding was applied during data acquisition and analysis. No statistical methods were used to predetermine sample size. Only male mice were used for experiments as the loss of the piRNA pathway does not effect any phenotype in female mice. Mouse embryos at E13.5, E14.5, E16.5 and E18.5, pups at P1 and P5, juveniles at P14 and adults between the ages of 9 and 12 weeks were used for experiments.

Animals were maintained at the University of Edinburgh, UK, in accordance with the regulation of the United Kingdom’s Home Office. Ethical approval for the UK mouse experimentation has been given by the University of Edinburgh’s Animal Welfare and Ethical Review Body and the work done under licence from the United Kingdom’s Home Office. Mice were housed under the following conditions: Lighting: 12-hour light to dark cycle from 7 AM to 7 PM; Temperature: 20-24 ºC; Humidity: 45-65 %; Caging: Tecniplast GM 500 Individual Ventilated Cages; Cage substrate: Aspen chips; Enrichment: Aspen chew sticks, carboard dome home, rodent roll and plastic tube for non-aversive handling

### Immunofluorescence and foci counting

Immunofluorescence (IF) experiments were done on freshly cryo-sectioned OCT-embedded E16.5 foetal testis or adult testis samples (6 μm sections) as previously described^23^. The following primary antibodies were used in this study – rabbit monoclonal anti-HA (clone C29F4, 3724, Cell Signaling Technologies, lot 12) 1:200, mouse monoclonal anti-HA (clone 6E2, 2367, Cell Signaling Technologies, lot 5) 1:100, rabbit polyclonal anti-LINE1-ORF1p 1:500 ^37^, rabbit polyclonal anti-IAP-GAG (a kind gift from B. Cullen, Duke University, Durham, NC, USA) 1:500, rabbit polyclonal anti-γH2AX (IHC-00059, Bethyl Laboratories, lot 5) 1:500, rabbit polyclonal anti-MIWI2 ^38^ 1:500, anti-SPOCD1 rabbit serum rb175 1:500 ^20^, anti-C19ORF84 rabbit serum rb632 1:500 ^20^, rabbit monoclonal anti-SPIN1 (clone E6R1Z, 8939S, Cell Signaling Technologies, lot 2) 1:100 (of a custom preparation of 1.1 μg/μl in PBS), rabbit polyclonal anti-TPR (ab84516, Abcam, lot 1028208-6) 1:300, rabbit monoclonal anti-DNMT3L (clone E1Y7Q, 13451S, Cell Signaling Technologies, lot 1) 1:500, mouse monoclonal anti-H3K9me3 (clone 2AG-6F12-H4, 39285, Active Motif, lot 31018002-11) 1:200, rabbit polyclonal anti-H3K4me3 (39159, Active Motif) 1:500, mouse monoclonal anti-H3K4me3 (mAbcam12209, Abcam, lot GR3253794-12) 1:100, rat monoclonal anti-HP1B (clone MAC353, ab10811, Abcam, lot GR3266755-6) 1:300. All primary incubations were performed overnight at 4 ºC. Secondary antibodies used were – donkey anti-rabbit IgG Alexa Fluor 488 (A-21206, Invitrogen) or 568 (A10042, Invitrogen), donkey anti-mouse IgG Alexa Fluor 568 (A10037, Invitrogen) or 647 (A-31571, Invitrogen), and donkey anti-rat IgG Alexa Fluor 647 (A48272, Invitrogen), all at a dilution of 1:1,000 in blocking buffer with 10 μg/ml DAPI. All secondary incubations were for 1 hour at room temperature.

Images were taken on a Zeiss LSM880 with Airyscan module in Super-Resolution (SR) mode, with optimal pinhole settings for SR imaging. The most restrictive excitation and emission filters available were chosen to prevent signal bleed-through between channels. Images were SR-processed with the Zeiss Zen 3.5 software “Airyscan processing” function with settings “3D” and a strength of “Auto”. Properly stained germ cells with normal oval morphology containing SPOCD1, C19ORF84 and SPIN1 foci in 1 foetal testis section were counted manually through the eyepiece at a Zeiss Airyscan 880 microscope using the 63x/1.4NA Plan-Apochromat objective, the same used for imaging. For judging overlap of foci with HP1B and H3K4me3, the maximum brightness of the corresponding channels was set to prevent saturation in ImageJ (version 1.54k, as part of Fiji 2.14.0) ^39^ and overlap in signal was assessed manually and representative profile plots were generated. Channel intensities were scaled to be between 0 and 1 based on minimum and maximum intensity on a per-channel basis for the profile plots.

### Quantitative co-localisation analysis

Images were acquired and Airyscan-processed as described. Germ cells were segmented using Cellpose-SAM ^40^ on the channel containing the nuclear piRNA pathway protein stain (version 1.1.1 of the plugin, https://github.com/COIL-Edinburgh/ROI_NucleusColocalisation/releases/tag/1.1.1). For MIWI2, which is both cytoplasmic and nuclear at E16.5, an additional segmentation step was added based on DAPI to select only germ cell nuclei at all time points (version 2.01 of the plugin, https://github.com/COIL-Edinburgh/ROI_NucleusColocalisation/releases/tag/2.01). Cellpose masks were passed into Coloc 2 (Fiji) ^39^ to calculate Pearson’s correlation coefficients after bisection thresholding with a point spread function of 4 and 20 rounds of Costes randomisations. Segmented cells were manually filtered to remove very damaged cells or germ cells which overlapped somatic cells. Cells containing foci were assigned by manual examination of the analysed images in ImageJ. Pearson’s R values above the channel intensity thresholds determined by the Coloc 2 bisection algorithm were plotted using custom Python scripts. All code utilised is available at the Github repository https://github.com/COIL-Edinburgh/ROI_NucleusColocalisation. A version of record has been deposited at Zenodo with DOI: 10.5281/zenodo.17200734.

### Protein purification

GST-3C-tagged mouse SPOCD1 TFIISM domain (amino acids 407-568) was cloned into a pET-based vector. GST-SPOCD1-TFIISM domain was expressed in *E. coli* BL21 (*DE3*)-pRIPL cells. Bacteria were grown in 1 L 2xYT media supplemented with 50 μg/ml kanamycin and 25 μg/ml chloramphenicol at 37 ºC until OD600 = 0.8 was reached. The temperature was then reduced to 16 ºC and 0.1 mM IPTG was added to induce expression. The bacteria were harvested after 20 hours and stored at -80 ºC until needed. Cell pellets were resuspended in lysis buffer (20 mM Tris-HCl pH 7.5, 500 mM NaCl, 4 mM MgCl_2_, 1 mM DTT, and cOmplete EDTA-free protease inhibitor (04693132001, Roche)). Resuspended pellets were lysed using the Constant Systems 1.1 kW TS Cell Disruptor with 1 pass at 25 kpsi, and Triton X-100 was added to a final concentration of 0.1 %. Lysates were clarified by centrifugation at 45,000 g for 40min. Cleared lysate was incubated with 3 ml Glutathione Sepharose High Performance (17-5279-02, Cytiva) beads pre-equilibrated in wash buffer (lysis buffer without protease inhibitor or Triton) for 2 hours at 4 ºC. After extensive washes with equilibration buffer, proteins were eluted with 10 mM reduced glutathione in wash buffer at pH 8.0. Pooled elutions were dialysed overnight at 4 ºC into 20 mM Tris-HCl pH 7.5, 150 mM NaCl, 4 mM MgCl_2_, 5 % glycerol and 1 mM DTT. Dialysed eluate was concentrated with a 30 kDa MWCO PES ultrafiltration device (88531, Pierce) to 4 mg/ml, aliquoted and flash-frozen in liquid nitrogen, and stored at -80 ºC until needed. GST-SPOCD1-TFIISM K464A was derived by PCR mutagenesis of the pET-GST-SPOCD1-TFIISM plasmid. The mutant protein was purified under the same conditions as the wild-type protein.

The previously characterised human TPR fragments ^28^, TPR-N (amino acids 1-774), TPR-M (amino acids 775-1700) and TPR-C (amino acids 1701-2353) were cloned into the same pET-based backbone as SPOCD1 TFIISM, with amino-terminal GST-3C tags. The initial steps to purify the TPR fragments were the same as for the SPOCD1 TFIISM domain. After washing, the beads were resuspended in 20 ml 3C cleavage buffer (20 mM Tris-HCl pH 7.5, 150 mM NaCl, 4 mM MgCl_2_, 5 % glycerol and 3 mM DTT) and 0.5 ml of home-made GST-tagged HRV 3C protease (0.5 mg/ml) was added. The cleavage proceeded overnight at 4 ºC on a rotator. After 18 hours, the supernatant was collected and subjected to 2 rounds of reverse-affinity with 1 ml and 0.5 ml freshly equilibrated Glutathione Sepharose resin for 1 hour each time at 4 ºC on a rotator. The supernatants were then concentrated to 1 mg/ml with 50 kDa MWCO PES concentrators (88541, Pierce), aliquoted and flash-frozen in liquid nitrogen and stored at -80 ºC until needed.

MBP-TPR (775-962) and MBP-GFP (monomeric super-folder/SiriusGFP) were cloned into the 9C plasmid (pET-His_6_-MBP-TEV, Addgene #48286). The cell pellets were obtained as above, except that bacteria were grown with 150 μg/ml ampicillin. Pellets were resuspended in lysis buffer (20 mM Tris-HCl pH 7.5, 150 mM NaCl, 4 mM MgCl_2_, 1 mM DTT, 50 mM Imidazole for MBP-TPR (775-962) or 10 mM imidazole for MBP-GFP, 10 μg/ml DNaseI (D4527, Sigma), and cOmplete EDTA-free protease inhibitor). Pellets were lysed and clarified as above. Clarified lysates were incubated with His-Select Ni-NTA agarose (P6611, Millipore) equilibrated in wash buffer (lysis buffer but with 500 mM NaCl and without DNase and Protease Inhibitor) at 4 ºC for 2 hours. After extensive washes with wash buffer, proteins were eluted with 100 mM, 200 mM and 300 mM imidazole in 3 fractions. The 100 mM imidazole fraction was concentrated to 1 mg/ml using 30 kDa MWCO PES concentrators, snap-frozen in liquid nitrogen and stored at -80 ºC until needed. MBP-TPR (775-962) E830A/E832A was derived by PCR mutagenesis of the 9C-MBP-TPR (775-962) plasmid and the mutant protein purified under the same conditions as the wild-type protein.

### Cell culture and transfection

Mouse embryonic stem cells (mESCs, E14Tg2a, a kind gift from Prof. Keisuke Kaji, University of Edinburgh) were cultured and transfected as described previously ^41^. Cells expressing SPOCD1-HA, SPOCD1(ΔTFIISM – Δ410-519)-HA or SPOCD1(ΔSPOC – Δ680-824)-HA or empty insert were obtained by random integration of a protein-of-interest-IRES-GFP cassette by a *PiggyBac* transposase system as described previously ^41^. For FACS gating strategies to sort GFP+ cells, see Supplemental Figure 2b. For experiments, 500,000 cells were seeded per well of a 6-well plate and grown overnight before pellets were collected by trypsinization. Pellets were washed once with PBS and snap-frozen in liquid nitrogen and stored at -80 ºC prior to use. HEK293T cells (O’Carroll laboratory stock) were cultured and transfected with 1 μg of each pcDNA3.1+ – FLAG-TPR fragment as previously described ^19^, with the addition of 100 U/ml penicillin/streptomycin (15140122, Gibco), 1X MEM Non-Essential Amino Acids (11140050, Gibco), and 0.1 mM β-mercaptoethanol (31350010, Gibco). Media was changed 16 hours post transfection and cells were collected as above at 42 hours post transfection. Cell lines were not additionally authenticated. All cells were regularly checked for mycoplasma contamination.

### Immunoprecipitation and mass spectrometry (IP-MS)

For the IP-MS of SPOCD1-HA and domain deletion mutants from mESCs, cell pellets from 1 well of a 6-well plate per replicate were lysed in hypotonic lysis buffer (10 mM Tris-HCl pH 8, 10 mM KCl, 5 mM MgCl_2_, 0.1 % IGEPAL CA-630, cOmplete™ ULTRA protease inhibitor EDTA-free (05892791001, Roche), 50 U/ml Benzonase™ (71206, Millipore)) with 20 strokes in a Tenbroeck glass dounce homogeniser (10428341, Fisher Scientific). Lysates were incubated for 30 minutes at 4 ºC and then cleared for 5 minutes at 21,000 g. Cleared lysates were added to 50 μl anti-HA magnetic beads (88837, Pierce, lot YD360611), additionally cross-linked with dimethyl pimelimidate (DMP) (21667, Thermo). mESCs transfected with empty vector were used as controls. Beads were washed 4 times with wash buffer (50 mM Tris pH 8, 100 mM KCl, 5 mM MgCl_2_, 0.1 % IGEPAL CA-630), then eluted with 0.1 % Rapigest (186001860, Waters). IP-MS analysis proceeded as described previously ^20^.

For anti-TPR and anti-SPOCD1 IP-MS from foetal testes, Protein G Dynabeads (10004D, Invitrogen) were crosslinked with rabbit anti-TPR antibody (ab84516, Abcam), rabbit serum 175 against SPOCD1 ^20^, or control commercial rabbit serum (NS01L, Sigma-Aldrich) in a 1:2, 2:3 or 2:3 serum to beads ratio, respectively, with 2 mM DMP in borate buffer pH 9. Anti-TPR IP-MS was performed using anti-TPR crosslinked to Protein G Dynabeads from 25 wild-type E16.5 foetal testes per replicate as described previously ^20^, with control commercial rabbit serum crosslinked to Protein G Dynabeads used for the control immunoprecipitation. Anti-SPOCD1 IP-MS was performed using anti-SPOCD1 rabbit serum rb175 crosslinked to Protein G Dynabeads from 24 wild-type and 24 *Spocd1*^*K464A*^ E16.5 foetal testes per replicate utilising mild lysis buffer (10 mM Tris-HCl pH 8, 100 mM KCl, 5 mM MgCl_2_, 0.1 % IGEPAL CA-630, cOmplete™ ULTRA protease inhibitor EDTA-free, 50 U/ml Benzonase™), with other conditions being the same as for the anti-TPR IP-MS. All statistically significant (P < 0.05) enriched (Enrichment > 4-fold) proteins in the anti-TPR IP-MS are shown in Supplementary Table 1. All statistically significant (P < 0.05) enriched (Enrichment > 4-fold with either wild-type SPOCD1 or SPOCD1-K464A) proteins in the anti-SPOCD1 IP-MS are shown in Supplementary Table 2.

### Pull-down assays

Pellets of 2 wells of six well plate HEK293T cells expressing Flag-tagged TPR fragments were lysed in 100 μl mild lysis buffer (20 mM Tris-HCl pH 7.5, 100 mM NaCl, 4 mM MgCl_2_, 3 mM DTT, 5 % glycerol, 0.5 % Triton X-100, cOmplete ULTRA EDTA-free protease inhibitor, 50 U/ml Benzonase™) by gentle pipetting. Lysis was allowed to continue for 30 minutes on a rotating wheel at 4 ºC. Lysates were then diluted 1:4 with lysis buffer without Triton X-100 to reduce Triton concentration to 0.1 %. Lysates were then clarified by centrifugation for 5 minutes at 21,000 g. Supernatant was then pre-cleared with 50 μl Glutathione Sepharose beads equilibrated with wash buffer (20 mM Tris-HCl pH 7.5, 100 mM NaCl, 4 mM MgCl_2_, 3 mM DTT, 5 % glycerol, 0.1 % Triton X-100) for a further 30 minutes on a rotating wheel at 4 ºC. 4 μg GST-SPOCD1-TFIISM or GST were then added to each lysate, and 5 % of the volume was collected as input. After 15 minutes on a rotating wheel at 4 ºC, 20 μl of Glutathione Sepharose beads were added and the incubation continued for 1 hour. Samples were then washed 6 times with 1 ml wash buffer before being eluted from the resin with 40 μl of 20mM reduced glutathione in wash buffer for 10 minutes at 4 ºC. Inputs and eluates were run on a Bolt™ 4-12 % Bis-Tris Plus gradient gel (NW04125BOX, Invitrogen) and transferred onto nitrocellulose membrane (Amersham Protran™ 0.45 NC, Cytiva) following manufacturer guidelines. The membrane was then incubated overnight with mouse anti-FLAG (clone M2, F1804, Invitrogen, 1:1,000, lot 261540), mouse anti-alpha tubulin (clone DM1A, T9026, Sigma, 1:10,000, lot 137585) and mouse anti-GST (clone 8-326, MA4-004, Invitrogen, 1:1,000, lot YK382039), all diluted in 4 % skimmed milk in TBS-T. Membranes were then washed 4 times for 5 minutes with TBS-T before being incubated with IRDye 800CW donkey anti-mouse IgG (926-32212, LI-COR Biosciences, 1:20,000) in TBS-T. Membranes were washed 3 times for 5 minutes with TBS-T before being imaged on a LI-COR Odyssey CLx imaging system. Exposure of entire images were adjusted in ImageJ and areas of interest were cropped for presentation.

For MBP pulldowns, 4 μg MBP-TPR (775-962) (wild-type or E830A/E832A) or MBP-GFP were mixed with 20 μg GST-SPOCD1-TFIISM (wild-type) or GST-SPOCD1-TFIISM (K464A) in binding buffer (20 mM Tris-HCl pH 7.5, 100 mM NaCl, 4 mM MgCl_2_, 3 mM DTT, 5 % glycerol, 0.02 % IGEPAL CA-630) in a volume of 250 μl. 10 % was collected as input and 15 μl of amylose resin (E8021S, NEB), equilibrated in binding buffer, was added. After 1 hour of incubation on a rotating wheel at 4 ºC, samples were washed 5 times with binding buffer before being eluted with 30 μl of 0.1 % SDS in 50 mM Tris-HCl pH 8.0 at 50 ºC for 10 minutes. Samples were analysed by SDS-PAGE and Coomassie Blue staining.

### Cross-linking mass spectrometry analysis

GST pull-down was performed with 21 μg GST-SPOCD1-TFIISM and 210 μg recombinant TPR-M fragment as described above, except that Tris-HCl pH 7.5 in the wash buffer was swapped for HEPES-NaOH pH 7.5. After 6 washes, beads were resuspended with 50 μl wash buffer. BS3 (bis(sulfosuccinimidyl)suberate) cross-linker (21580, Thermo-Fisher) dissolved in wash buffer was added to the beads at BS3:protein mass ratios of 2:1 and 4:1. Reactions were allowed to proceed for 2 hours on ice, then terminated by the addition of 2 μl of 2.0 M ammonium bicarbonate (ABC). Half the sample was subjected to SDS-PAGE to confirm cross-linking, and the other half was used for on-beads digestion. Cross-linking buffer was removed, and the beads resuspended in 60 μl of denaturation buffer (8 M urea in 50 mM ABC, pH 8.0). DTT was added to 10 mM and incubated for 30 minutes at room temperature. Iodoacetamide was then added to 55 mM working concentration and incubated for 20 minutes at room temperature in the dark. Endoproteinase Lys-C (VA1170, Promega) was added to a final concentration of 15 ng/μl, and digestion proceeded for 4 hours at room temperature. Samples were diluted 4x with 50 mM ABC (final volume ~360 μl) and trypsin (90057, Pierce) was added to a final concentration of 5 ng/μl and digestion proceeded overnight at room temperature. Tryptic digestion was stopped by acidifying the samples to pH <2.5 with 10 % trifluoracetic acid. Supernatants were then desalted with C18 STAGE tips as described previously for IP-MS ^42^. LC-MS/MS analysis was performed using Orbitrap Fusion Lumos (Thermo-Fisher Scientific) with a “high/high” acquisition strategy as described previously ^4^. Mass-spectrometry data were analysed using ProteoWizard (version 3.0) ^43^ and the Xi suite (version 1.7.6.4) ^44^, and only auto-validated heteromeric (between proteins) cross-linked peptides were considered. Identified cross-links underlying Fig. 2e are shown in Supplementary Table 3.

### Protein conservation and structure visualization

The mouse SPOCD1 TFIIS-M structure was generated using AlphaFold2 ^45^ using the ColabFold v1.5.5 notebook ^46^. Images were generated using PyMol (version 3.1.3.1, Schrodinger LLC). The surface charge was calculated with APBS ^47^, and sequence conservation was determined with ConSurf ^48^. Multiple sequence alignments for SPOCD1 was generated with Clustal Omega ^49^ and visualised in JalView (2.11.4.1) ^50^ for the canonical SPOCD1 sequences from UniProt ^51^ for the following species – Mouse (*Mus musculus*, B1ASB6), Golden hamster (*Mesocricetus auratus*, A0A3Q0D6B7), Ord’s kangaroo rat (*Dipodomys ordii*, A0A1S3FIT4), Western European hedgehog (*Erinaceus europaeus*, A0A1S3WPZ3), Rabbit (*Oryctolagus cuniculus*, G1SPR0), Aardvark (*Orycteropus afer afer*, A0A8B7AXN8), Bison (*Bison bison bison*, A0A6P3HA20), Bovine (Bos taurus, F1MG39), Goat (*Capra hircus*, A0A452FMH8), Sheep (*Ovis aries*, W5NRM3), Pig (*Sus scrofa*, F1SV96), Horse (*Equus caballus*, F6YBJ1), Alpaca (*Vicugna pacos*, A0A6J3AYV9), California sealion (*Zalophus californianus*, A0A6J2C2W2), Northern fur seal (*Callorhinus ursinus*, A0A3Q7MZA7), Atlantic bottle-nosed dolphin (*Tursiops truncatus*, A0A6J3PXS9), Sperm whale (*Physeter macrocephalus*, A0A455B8T1), Blue whale (*Balaenoptera musculus*, A0A8B8W162), Great Himalayan leaf-nosed bat (*Hipposideros armiger*, A0A8B7SLV8), Large flying fox (*Pteropus vampyrus*, A0A6P3Q928), Leopard (*Panthera pardus*, A0A6P4UE11), Cat (*Felis catus*, A0A5F5XDK8), Red fox (*Vulpes vulpes*, A0A3Q7T0D7), Dog (*Canis lupus familiaris*, A0A8P0P5S7), Polar bear (*Ursus maritimus*, A0A8M1EZU5), Greater bamboo lemur (*Prolemur simus*, A0A8C8YEI0), Giant panda (*Ailuropoda melanoleuca*, G1MHH0), Rhesus macaque (*Macaca mulatta*, F7G2T4), Northern white-cheeked gibbon (*Nomascus leucogenys*, G1QNN7), Sumatran orangutan (*Pongo abelii*, H2N866), Gorilla (*Gorilla gorilla gorilla*, G3RKR7), Chimpanzee (*Pan troglodytes*, H2R1B9), Human (*Homo spaiens*, Q6ZMY3), American alligator (*Alligator mississippiensis*, A0A151MMW3), Chinese alligator (*Alligator sinensis*, A0A1U8CWC7), Snapping turtle (*Chelydra serpentina*, A0A8T1SPX3), Central bearded dragon (*Pogona vitticeps*, A0A6J0UYI0), Mainland tiger snake (*Notechis scutatus*, A0A6J1UEZ9), Indian cobra (*Naja naja*, A0A8C6YBU2), American chameleon (*Anolis carolinensis*, H9GI50), Western clawed frog (*Xenopus tropicalis*, A0A8J0T0T7), African clawed frog (*Xenopus laevis*, A0A1L8HFK1).

### Western blotting of foetal testes

Foetal testis protein extracts for western blot analysis were made by lysing one E16.5 foetal testis of the indicated genotype (per replicate) in mild lysis buffer (150 mM NaCl, 2.5 mM MgCl_2_, 50 mM Tris-HCl pH 8, 0.5 % Triton X-100, cOmplete ULTRA EDTA-free protease inhibitor, 50 U/ml Benzonase™) using micro-pestles for tissue homogenization (AXYPES15BSI, Corning). Lysates were cleared by centrifugation for 5 minutes at 21,000 g. SDS-PAGE and western blotting were performed as described above, except the transfer was done overnight. After blocking, the blot was cut into 4 sections based on the molecular weight marker (<75 kDa, 75 kDa to 110 kDa, 110 kDa to 180 kDa, >180 kDa) The <75 kDa part was stained with mouse anti-alpha-tubulin (T9026, Sigma, 1:1,000), while the 75-110 kDa part was probed with rabbit anti-MIWI2 ^38^ (1:1,000), the 110-180 kDa part with rabbit anti-SPOCD1 rb175 (^20^, 1:1,000) and the >180 kDa part with rabbit anti-TPR (ab84516, Abcam, 1:1,000). All primary incubations were done overnight at 4 ºC in blocking buffer. IRDye 680RD donkey anti-mouse IgG (926-68072, LICOR Biosciences) and IRDye 800CW donkey anti-rabbit IgG (926-32213, LICOR Biosciences) secondary antibodies were used at 1:10,000 in blocking buffer at room temperature for 1 hour, and the blots were imaged on a Chemidoc MP imager (Bio-Rad). Exposure of the whole blots was adjusted for presentation with ImageJ.

### Histology of mouse samples

Histology experiments on mouse samples were done as previously described ^19^. Acquired images were white-balanced and zooms and crops of regions of interest were obtained using QuPath (0.5.1) ^52^.

### Terminal deoxynucleotidyl transferase dUTP nick end labeling (TUNEL) assay

TUNEL assay experiments were done as previously described ^19^.

### Fluorescence-activated cell sorting (FACS)

For Enzymatic Methylation (EM)-seq, CD9^+^ c-Kit^–^ spermatogonia were isolated from postnatal day 14 testes as described previously ^32^ using Fc block (anti-mouse CD16/32) (clone 93, 14-0161-86, eBioscience, lot 2297433) 1:50, biotin-conjugated anti-CD45 (clone 30-F11, 13-0451-85, eBioscience, lot 2349865) 1:400, biotin-conjugated anti-CD51 (clone RMV-7, 104104, Biolegend, lot B308465) 1:100 anti-CD9^APC^ (clone eBioKMC8, 17-0091-82, eBioscience, lot 2450733) 1:200, anti-cKit^PE-Cy7^ (clone 2B8, 25-1171-82, eBioscience, lot 2191977) 1:1600, streptavidin^V450^ (560797, BD bioscience, lot 1354158) 1:400, and 1 μg/ml DAPI. For gating strategies, see Supplemental Figure 2a.

mESCs with stable integration of the *piggyBac* cassette were isolated based on GFP reporter expression. For FACS gating strategies to sort GFP+ cells, see Supplemental Figure 2b.

### Whole-genome methylation sequencing and analysis

Whole genome methylation sequencing experiments and analysis were done as previously described ^19^ with data for *Spocd1*^*-/-*^, *Miwi2*^*-/-*^ and corresponding wild-type samples retrieved from ArrayExpress: E-MTAB-7997 and *C19orf84*^*-/-*^ samples from ArrayExpress: E-MTAB-11612 ^20^. Analysis of EM-Seq data was done as described previously using the Trim Galore (0.6.7), cutadapt (1.18) and Bismarck (v0.23.0) software packages ^19^. Median methylation and variance in methylation across replicates were assessed using the CpG methylation percentage of each transposon locus in the LINE1 families L1Md_Gf, L1Md_T, L1Md_A and L1Md_F, and only 3 of 4 *Spocd1*^*+/+*^ replicates were considered for consistency with other genotypes having 3 replicates. Scripts relating to EM-Seq analyses are available at https://github.com/tamchow/spocd1_pirna-directed-dna-met-variance/ (version of record deposited at Zenodo, DOI: 10.5281/zenodo.17162836) and https://github.com/rberrens/SPOCD1-piRNA_directed_DNA_met (version of record deposited at Zenodo, DOI: 10.5281/zenodo.10509247).

### DNA fluorescence in-situ hybridisation (DNA-FISH) and heterochromatic loci analysis

E16.5 *Spocd1*^*+/+*^ and *Spocd1*^*K464A*^ foetal testis paraffin sections were prepared as described previously ^3^. Sections on slides were deparaffinised at 60 ºC for 20 minutes, 2x 10-minute xylene rinses, followed by a decreasing graded alcohol series into distilled water. Sections were microwaved fully covered with 0.1 M pH 6.0 sodium citrate buffer for 30 minutes and allowed to cool in the buffer for 20 minutes before being immersed in distilled water. Sections were briefly rinsed in 2x SSC, incubated for 5 minutes in 2x SSC at 75 ºC and denatured in 70 % formamide/2x SSC pH 7.5 for 3 minutes at 75 ºC. Sections were then plunged into ice cold 70 % ethanol for 2 minutes then through 90 % and 70 % ethanol before air drying. 1 μg fosmid probes (*Rasgrf1*: WI1-2719E16 – chr9:89858022-89895107, and control: WI1-447D3 – chr9:21644745-21687364, BacPac Genomics) were labelled by nick-translation to incorporate fluorescent dUTP (ChromaTide™ Alexa Fluor™ 594-5-dUTP, C11400, Thermo-Fisher) or digoxigenin dUTP (Digoxigenin-11-dUTP alkali-stable, 11093088910, Merck). 100ng of each fosmid, 6 μg of mouse Cot-1 DNA (Invitrogen, 18440016), and 5 μg of sonicated salmon sperm DNA (31149-10G-F, Sigma) were ethanol precipitated in a Spin-Vac and reconstituted in 15 μl of hybridisation mix (50 % formamide, 1 % Tween 20, and 10 % dextran sulphate in 2× SSC). Fosmid probes were denatured at 70 ºC for 5 minutes and reannealed at 37 ºC for 15 minutes, then hybridised overnight at 37 ºC to slides under a sealed coverslip. Slides were washed the next day for 4x 3 minutes in 2x SSC at 45 °C, 4x 3 minutes in 0.1x SSC at 60 °C. Following washes, digoxigenin-labelled probes were detected with fluorescein-sheep anti-digoxigenin Fab fragments (1:200; 11207741910, Merck, lot 70720200) and fluorescein-rabbit anti-sheep IgG (1:200; FI-6000, Vector Labs, lot W0811). Slides were washed with 4x SSC/0.1 % Tween-20 after each antibody incubation. Slides were washed with PBS and then stained with DAPI at 50 ng/ml in PBS, mounted in Vectashield (Vector Labs) and sealed with nail varnish. Fluorescence images were acquired using a Photometrics Prime BSI CMOS camera (Photometrics, Tuscon, AZ) fitted to a Zeiss AxioImager M2 microscope with the 63x/1.4NA Plan-Apochromat oil-immersion objective. Images were captured and deconvolved using a calculated point spread function with the constrained iterative algorithm of Zeiss Zen 3.5 software.

For manual assessment overlap of FISH’ed loci with heterochromatin, DAPI channel brightness was set to saturate chromocenters in somatic cells, and FISH’ed channel brightness was set to remove non-specific background until only specific foci were visible in ImageJ. A locus was considered heterochromatic if it completely or partially overlapped a region within a germ cell nucleus which was saturated for DAPI intensity as set prior. Images were anonymised before assessment by a person who did not perform the staining.

### DNA-FISH coupled with immunofluorescence

DNA-FISH was performed on 6 μm cryosections from wild-type and *Spocd1*^*K464A*^ E16.5 foetal testes. Sections were washed with PBS 2 times for 2 minutes each, fixed with 4 % PFA in PBS for 10 minutes at room temperature, then washed again with PBS two times for 2 minutes each. Sections were then permeabilised with 0.1 % Triton X-100 in PBS for 10 minutes and washed with PBS two times for 2 minutes each. After washing, slides were washed briefly with 2x SSC. Cellular RNA was degraded by incubating the sections with 100 μg/ml RNase A in PBS for 1 hour at 37 °C. Slides were then briefly washed twice with 2x SSC, dehydrated with a 70 %, 90 % and 100 % ethanol series for 2 minutes each, then air-dried at room temperature. DNA in the sections was denatured by heating the slides in a 70 °C oven for 5 minutes and then by incubating in a solution of 70 % formamide in 2x SSC (pH 7.5) for 15 minutes at 80 °C, following which the slides were air-dried.

LINE1 probe was produced by nick-translation to incorporate fluorescent dUTP (ChromaTide™ Alexa Fluor™ 594-5-dUTP, C11400, Thermo-Fisher) into a full-length L1MdTf_I element amplified by PCR from *Mus musculus* C57Bl/6N genomic DNA (mm39 genome coordinates chr3:55509814-55517839 on the antisense strand) cloned into the vector pMD20. 100 ng of pMD20-LINE1 probe and 5 μg of sonicated salmon sperm DNA (31149-10G-F, Sigma) were ethanol precipitated in a Spin-Vac and reconstituted in 15 μl of hybridisation mix. Probes were denatured at 70 ºC for 5 minutes and snap-chilled on ice. Probe hybridization and washing proceeded as described previously for *Rasgrf1* FISH.

Post-FISH, the slides were washed with PBS three times for 2 minutes each. Slides were then blocked with blocking buffer (PBS with 1 % w/v BSA, 10 % goat serum (Gibco, PCN5000) and 0.1 % Tween-20). SPOCD1 was detected with rabbit serum rb175 against SPOCD1 (1:200 in blocking buffer) overnight at 4 °C, washed with 3 times for 5 minutes each with PBS, then the primary antibody was detected with goat anti-rabbit IgG H+L conjugated to Alexa Fluor 647 (1:1000 in blocking buffer) (Invitrogen, A-21245, lot unknown) at room temperature for 1 hour. Slides were washed three times for 5 minutes each with PBS. DNA was stained with 1 μg/ml DAPI in PBS for 15 minutes at room temperature, briefly washed twice with PBS and rinsed in ultrapure water before mounting in Vectashield non-setting mounting medium. Coverslips were sealed with nail varnish prior to imaging.

Slides were imaged on a Zeiss LSM980 with Airyscan II module. Alexa Fluor 594 was excited with the 561 nm laser and detected with a 570-630 nm bandpass filter to prevent cross-excitation and detection of Alexa Fluor 647, which was excited with the 639 nm laser and detected with a 660 nm longpass filter. Images with at least 5 z-planes were acquired in Airyscan super-resolution (SR) mode and 3D Airyscan processed using the Zeiss Zen 3.5 software with auto settings.

Cells with SPOCD1-K464A foci were identified manually in ImageJ. The maximum brightness of the corresponding channels was set to prevent saturation in ImageJ and overlap in signal was assessed manually along with automated calculation of Pearson’ correlation coefficients using version 1.1.1 of the plugin.

### Quantification and statistical analysis

Data were plotted in R (version 4.4.2 (2024-06-14)) using the ggplot2, tidyr, dplyr, ggpubr and Hmisc toolkits (versions ggplot2_3.5.1, tidyr_1.3.1, dplyr_1.1.4, ggpubr_0.6.0, Hmisc_5.2.1), Python (version 3.12.9) using the pandas, scipy, scikit-learn, matplotlib and seaborn packages (versions pandas_2.2.3, scipy_1.14.1, scikit-learn_1.5.2, matplotlib_3.9.2, seaborn_0.13.2) or Microsoft Excel for Mac (Office 365, version 16.9). For the IP-MS data, statistical testing was performed with R (version 4.0.3 (2020-10-10)) using the R Studio software and with Perseus (version 1.6.5.0) ^53^. The CL-MS data was analysed with ProteoWizard (version 3.0) ^43^ and the Xi suite (version 1.7.6.4) ^44^. Unpaired, two-tailed Student’s t-tests were used to compare differences between groups and adjusted for multiple testing using Benjamini–Hochberg correction where indicated. Averaged data are presented as mean ± SEM (standard error of the mean) for comparisons or mean ± SD (standard deviation) for descriptive statistics, unless otherwise indicated. No statistical methods were used to predetermine sample size. The experiments were not randomized, and the investigators were not blinded to allocation during experiments and outcome assessment unless otherwise noted.

## Extended Data

**Extended Data Figure 1:**
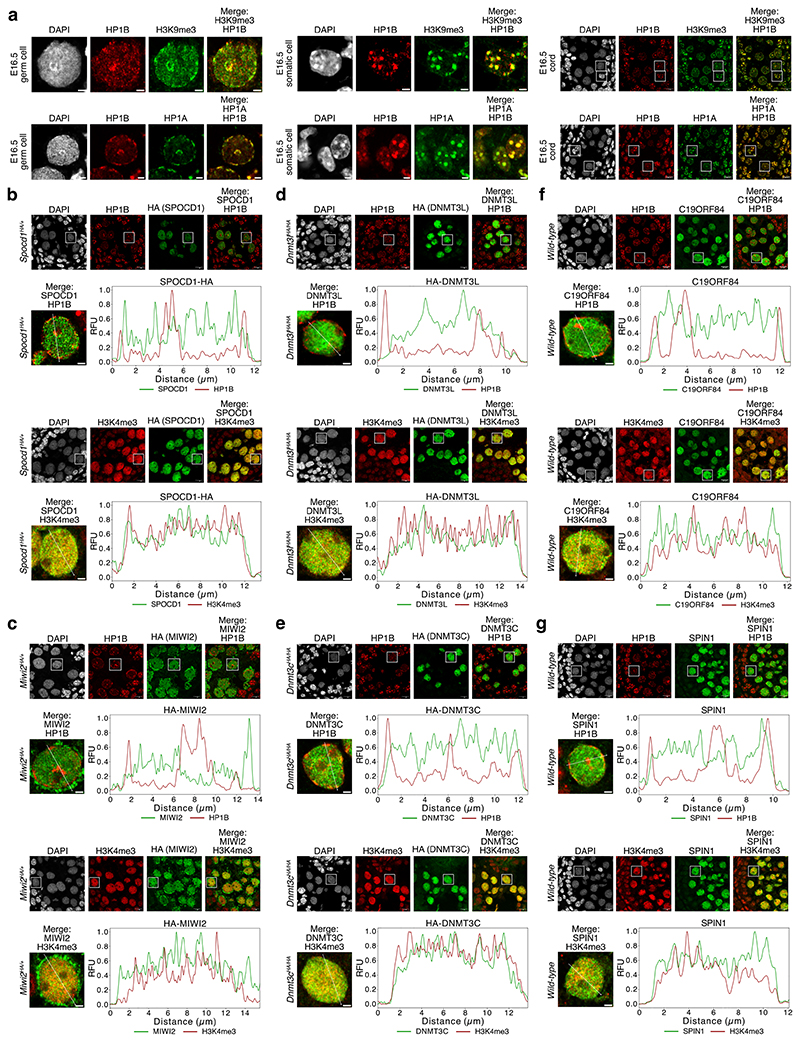
Nuclear piRNA factors and the *de novo* methylation machinery are euchromatic **a**, Representative E16.5 gonocyte, somatic cell and corresponding seminiferous cord stained for DAPI, HP1B (red) and H3K9me3 (green, top row) or HP1A (green, bottom row) in foetal testis sections from wild-type mice. Scale bars, 2 μm (top row), 10 μm (bottom row). White rectangles on images of seminiferous cords highlight cells shown zoomed-in. **b-e**, Representative E16.5 seminiferous cord stained for HA (green), HP1B (red, top row) or H3K4me3 (red, bottom row) and DAPI (blue) in foetal testis sections from *Spocd1*^*HA/+*^ (**b**), *Miwi2*^*HA/+*^ (**c**), *Dnmt3l*^*HA/HA*^ (**d**) and *Dnmt3c*^*HA/HA*^ (**e**) mice. **f-g**, Representative E16.5 seminiferous cord stained for C19ORF84 (**f**) or SPIN1 (**g**) (both green), HP1B (red, top row) or H3K4me3 (red, bottom row) and DAPI (blue) in foetal testis sections from wild-type mice. Also shown in **b-g** are the channel intensity profiles (RFU: Relative Fluorescence Units) along the indicated line passing through major heterochromatin domains (for HP1B) or avoiding nucleoli (for H3K4me3) for the representative gonocytes presented in Fig. 1. Images shown in both rows of **f** are from the same quadruple stain (DAPI, C19ORF84, H3K4me3 and HP1B) of a seminiferous cord. Images shown in both rows of **g** are from another quadruple stain (DAPI, SPIN1, H3K4me3 and HP1B) of a seminiferous cord. Images in **a** through **g** are representative of n = 4 (**b, c, e**) or n = 3 (**a, d, f, g**) biological replicates; scale bars, 10 μm for seminiferous cords and 2 μm for single gonocytes. White rectangles highlight cells shown in Fig. 1 and in the profile plots.

**Extended Data Figure 2:**
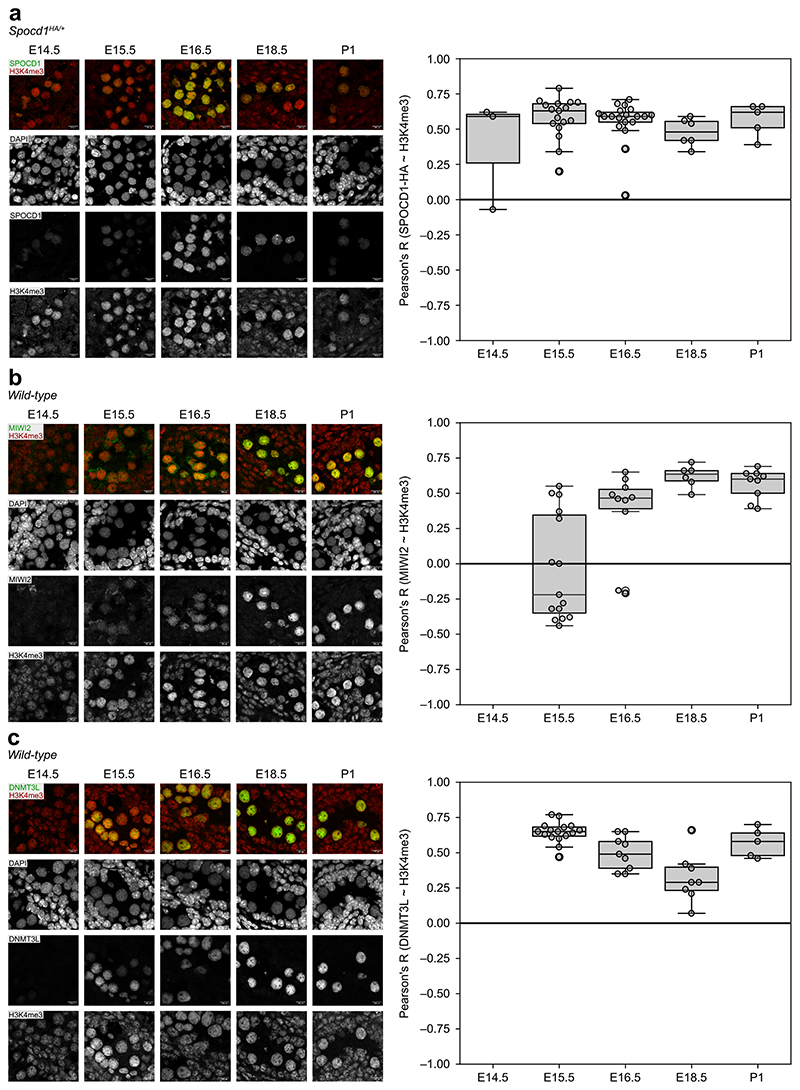
piRNA pathway factors are euchromatic from initiation of expression. **a-c**, Representative seminiferous cords stained for HA (SPOCD1-HA) (**a**), MIWI2 (**b**) and DNMT3L (**c**), H3K4me3 and DAPI from *Spocd1*^*HA/+*^ (**a**) or wild-type mice (**b-c**) at the indicated developmental stages. Shown to the right are boxplots of Pearson’s correlation coefficients of SPOCD1 (**a**), MIWI2 (**b**) and DNMT3L (**c**) with H3K4me3 in gonocyte nuclei at the indicated developmental stages. MIWI2 and DNMT3L are not expressed at E14.5, so co-localisation was not calculated for this developmental stage. Scale bars, 10 μm. Images in **a** through **c** are representative of n = 3 biological replicates. Box indicates interquartile range, central line represents the mean, and whiskers extend to median ± 1.5× the interquartile range or data limits, whichever is smaller.

**Extended Data Figure 3:**
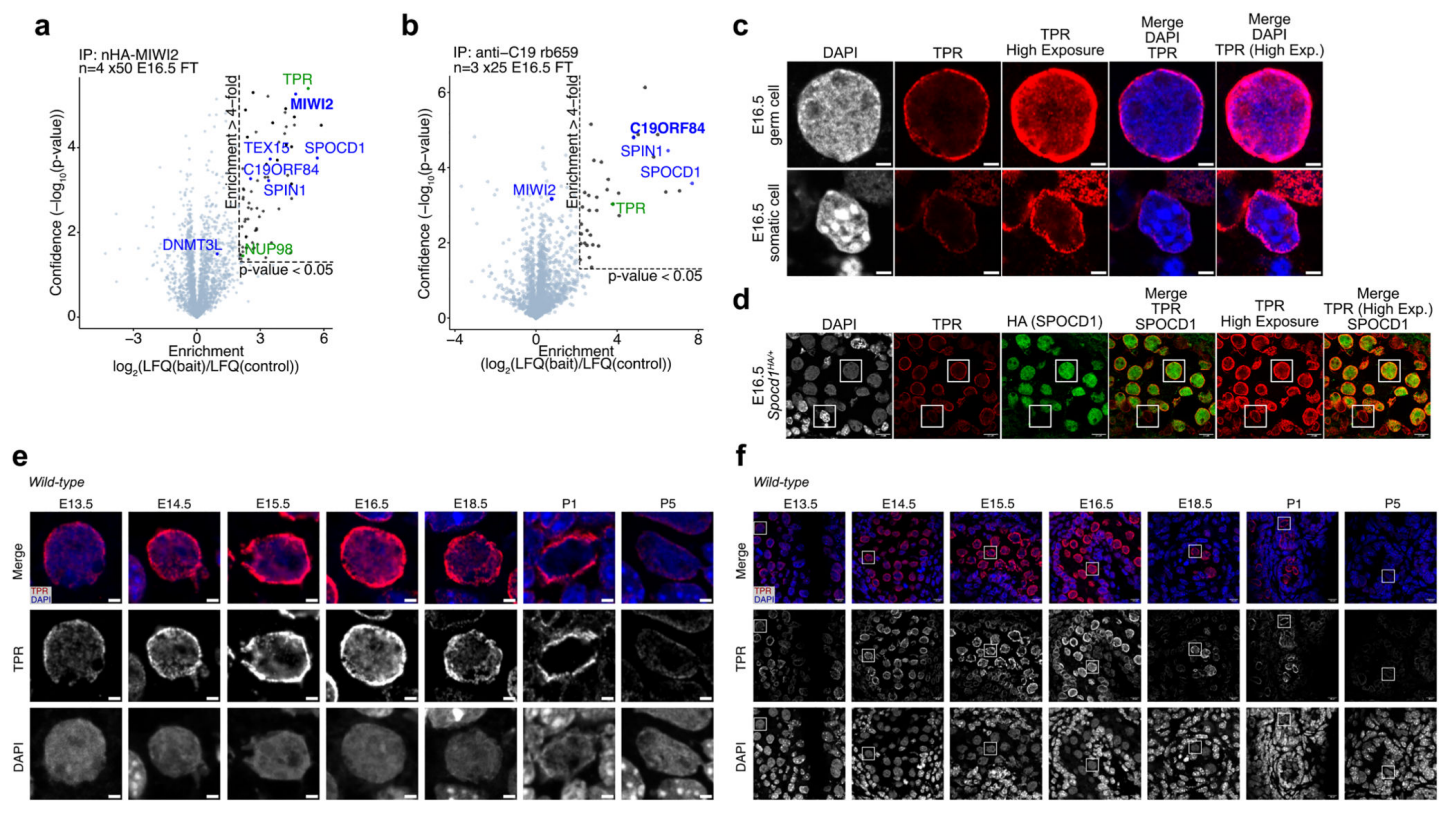
TPR is highly expressed in male foetal germ cells undergoing *de novo* genome methylation **a**, Volcano plot showing enrichment (log_2_(mean LFQ ratio of anti-HA immunoprecipitates from *Miwi2*^*HA/HA*^/wild-type E16.5 foetal testis lysates) and statistical confidence of proteins co-purifying with HA-MIWI2 (n = 4 with 50 testes per replicate, from previously published data ^19^). **b**, Volcano plot showing enrichment (log_2_(mean LFQ ratio of anti-C19ORF84 immunoprecipitated/anti-rabbit serum immunoprecipitated from wild-type E16.5 foetal testis lysates) and statistical confidence of proteins co-purifying with C19ORF84 (n = 3 with 25 testes per replicate, from previously published data ^20^). **c**, Single representative E16.5 germ and somatic cells stained for TPR (red) and DAPI (blue) in *Spocd1*^*HA/+*^ foetal testis sections. Scale bars, 2 μm. **d**, Representative E16.5 seminiferous cord stained for HA (green), TPR (red) and DAPI in foetal testis sections from *Spocd1*^*HA/+*^ mice. White rectangles highlight cells shown in Fig. 2f and Extended Data Fig. 3c. Scale bars, 10 μm. **e**, Representative gonocytes stained for TPR (red) and DAPI (blue) from wild-type mice at the indicated developmental stages. Scale bars, 2 μm. **e**, Representative seminiferous cords stained for TPR (red) and DAPI (blue) from wild-type mice at the indicated developmental stages. White rectangles highlight cells shown in Extended Data Fig. 3e. Scale bars, 10 μm. Images in **c** through **f** are representative of n = 3 biological replicates.

**Extended Data Figure 4:**
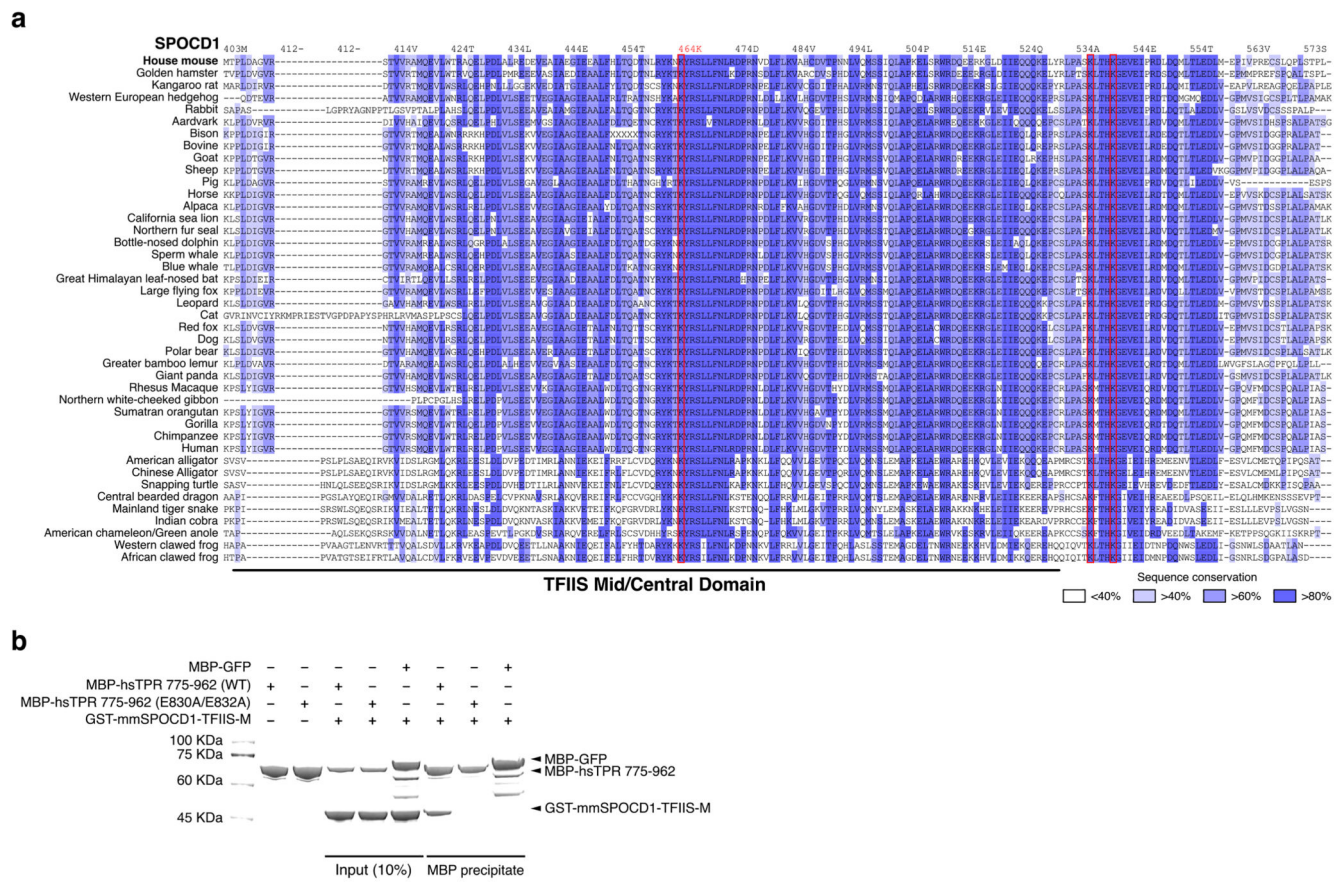
SPOCD1-K464A does not interact with TPR **a**, Multiple sequence alignment of the SPOCD1 TFIIS-M domains from the indicated species. Red rectangles highlight lysine residues making direct crosslinks with TPR-M in the CL-MS data. Sequence identity conservation is shown by depth of colour. **b**, Representative Coomassie-stained gel image of n = 3 MBP pull-down experiments with the indicated recombinant mouse SPOCD1 and human TPR fragments. For uncropped source gel image, see Supplementary Fig. 1d.

**Extended Data Figure 5:**
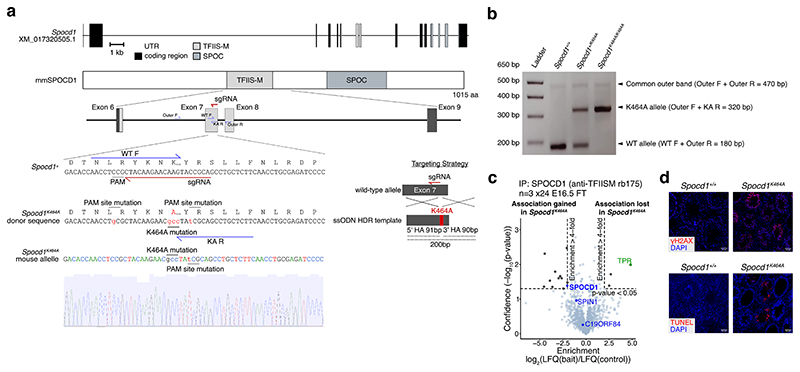
The *Spocd1*^*K464A*^ mouse allele **a**, Schematic representations of the mouse *Spocd1* locus and encoded 1015 amino acid protein are shown, along with a schematic of the CRISPR targeting strategy showing the location of single-stranded oligo DNA donor (ssODN) and homology arms (HA) used. The sgRNA used for generation of the *Spocd1*^*K464A*^ allele (red), adjacent PAM sites (red) and the primers used for genotyping (blue) are indicated, along with a representative sequencing trace of *Spocd1*^*K464A*^ exon 7 harbouring the 3bp mutation encoding the K464A mutation (highlighted in red). Sequencing was performed on n = 3 F_1_ animals. **b**, Representative image of PCR genotyping result for *Spocd1*^*+/+*^, *Spocd1*^*+/K464A*^ and *Spocd1*^*K464A*^ mice. PCR genotyping was performed for over 600 mice with similar results. **c**, Volcano plot showing enrichment (LFQ ratio of anti-SPOCD1 immunoprecipitates from wild-type/*Spocd1*^*K464A*^ E16.5 foetal testis lysates) and statistical confidence (−log_10_(P-value of two-sided Student’s t-test)) of proteins co-purifying with wild-type SPOCD1 (right quadrant) or SPOCD1-K464A (left quadrant) (n = 3 with 24 foetal testes per replicate per genotype). All identified proteins meeting the enrichment cut-off are listed in Supplementary Table 2. **d**, Representative adult testis sections of n = 3 wild type, *Spocd1*^*K464A*^ and *Spocd1*^*-/-*^ stained in blue for DAPI and red for the DNA damage marker γH2AX (top) or apoptotic cells by terminal deoxynucleotidyl transferase dUTP nick end labelling (TUNEL) assay (bottom). Scale bars, 50 μm.

**Extended Data Figure 6:**
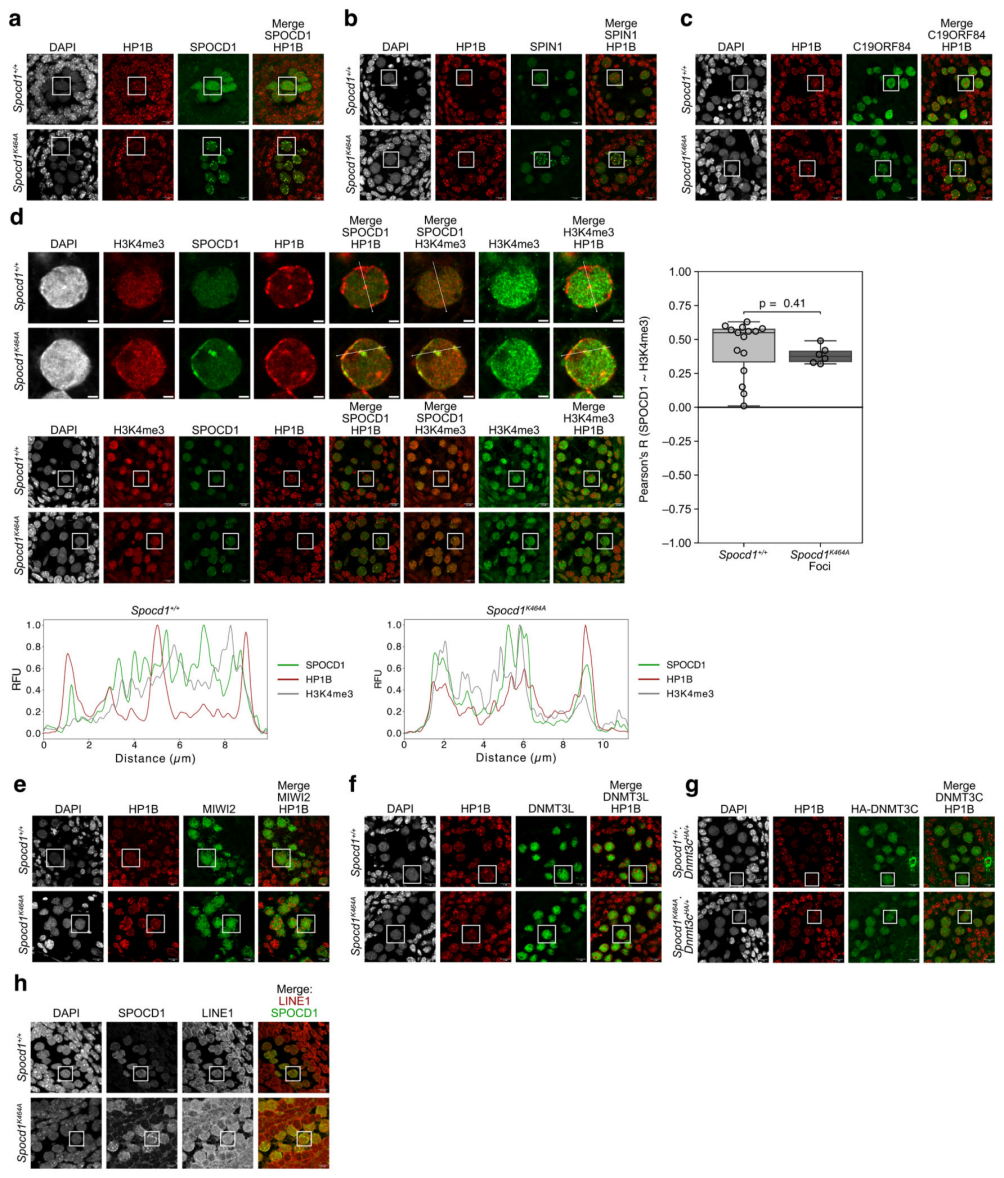
The SPOCD1-TPR interaction ensures that SPOCD1-SPIN1-C19ORF84 avoids heterochromatin **a-c, e-g**, Representative E16.5 seminiferous cord stained for DAPI, HP1B (red) and SPOCD1 (**a**), SPIN1 (**b**), C19ORF84 (**c**), MIWI2 (**e**), DNMT3L (**f**), or HA (HA-DNMT3C) (**g**) (all green) stains in wild-type (top row) or *Spocd1*^*K464A*^ (bottom row) foetal testis sections from littermates. Images are representative of n = 8 (**a**), n = 4 (**c**) or n = 3 (**b, d-f**) biological replicates of each genotype. White rectangles highlight cells shown zoomed-in in Fig. 4a-f. **d**, Representative E16.5 gonocyte (top two rows) and seminiferous cords (bottom two rows) stained for DAPI (blue), H3K4me3 (red or green), SPOCD1 (green) and HP1B (red) stains in wild-type (first and third row) or *Spocd1*^*K464A*^ (second and last row) foetal testis sections from littermates. Images are representative of n = 4 biological replicates of each genotype. White rectangles in the last two rows highlight cells shown in the first two rows. Also shown below are channel intensity profiles (RFU: Relative Fluorescence Units) along the indicated line passing through major heterochromatic domains in the representative gonocyte. Shown to the right are boxplots of Pearson’s correlation coefficients of SPOCD1 with H3K4me3 in gonocyte nuclei of the indicated genotypes. Box indicates interquartile range, central line represents the mean, and whiskers extend to median ± 1.5× the interquartile range or data limits, whichever is smaller. P-values are from unpaired, two-tailed Student’s T-tests. **h**, Representative E16.5 seminiferous cord stained for DAPI, SPOCD1 and LINE1 DNA in foetal testis sections of the indicated genotypes. Images are representative of n = 6 biological replicates of each genotype. White rectangles highlight cells shown in Fig. 4g. **a-h**, Scale bars, 10 μm for seminiferous cords and 2 μm for single gonocytes.

**Extended Data Figure 7:**
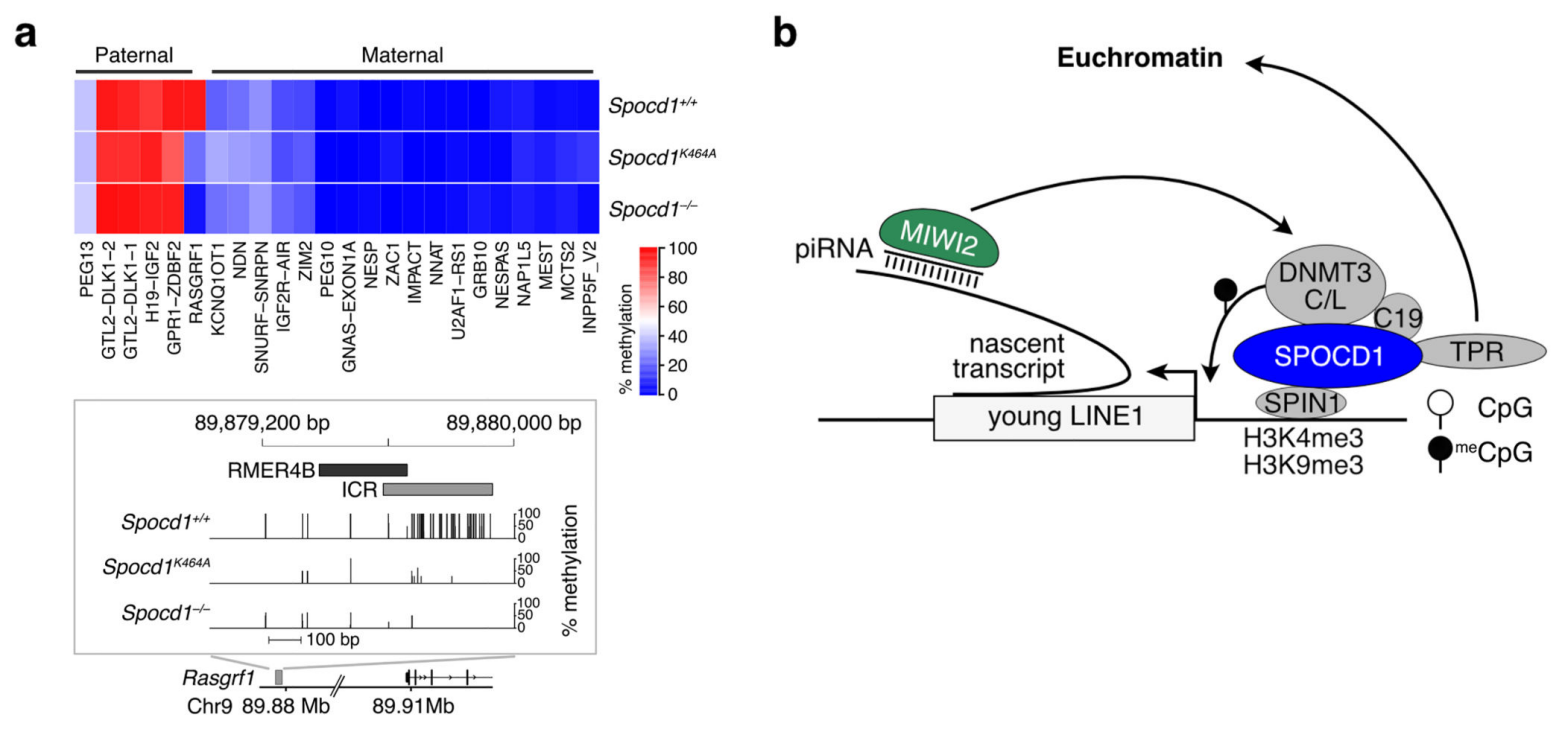
The SPOCD1-TPR interaction is necessary for *Rasgrf1* methylation **a**, Heatmap presentation of mean CpG methylation of imprinted loci in P14 undifferentiated spermatogonia of the indicated genotypes. The imprint control region (ICR) of *Rasgrf1* is shown in detail on the bottom. **b**, Cartoon representation of piRNA-directed epigenetic silencing of LINE1 elements in mice. TPR ensures SPOCD1-targeted LINE1 loci are present in euchromatin and accessible to the piRNA-directed DNA methylation machinery.

## Supplementary Material

Source Data Extended Data Figure 1

Source Data Extended Data Figure 2

Source Data Extended Data Figure 5

Source Data Extended Data Figure 6

Source Data Extended Data Figure 7

**Figure 1:**
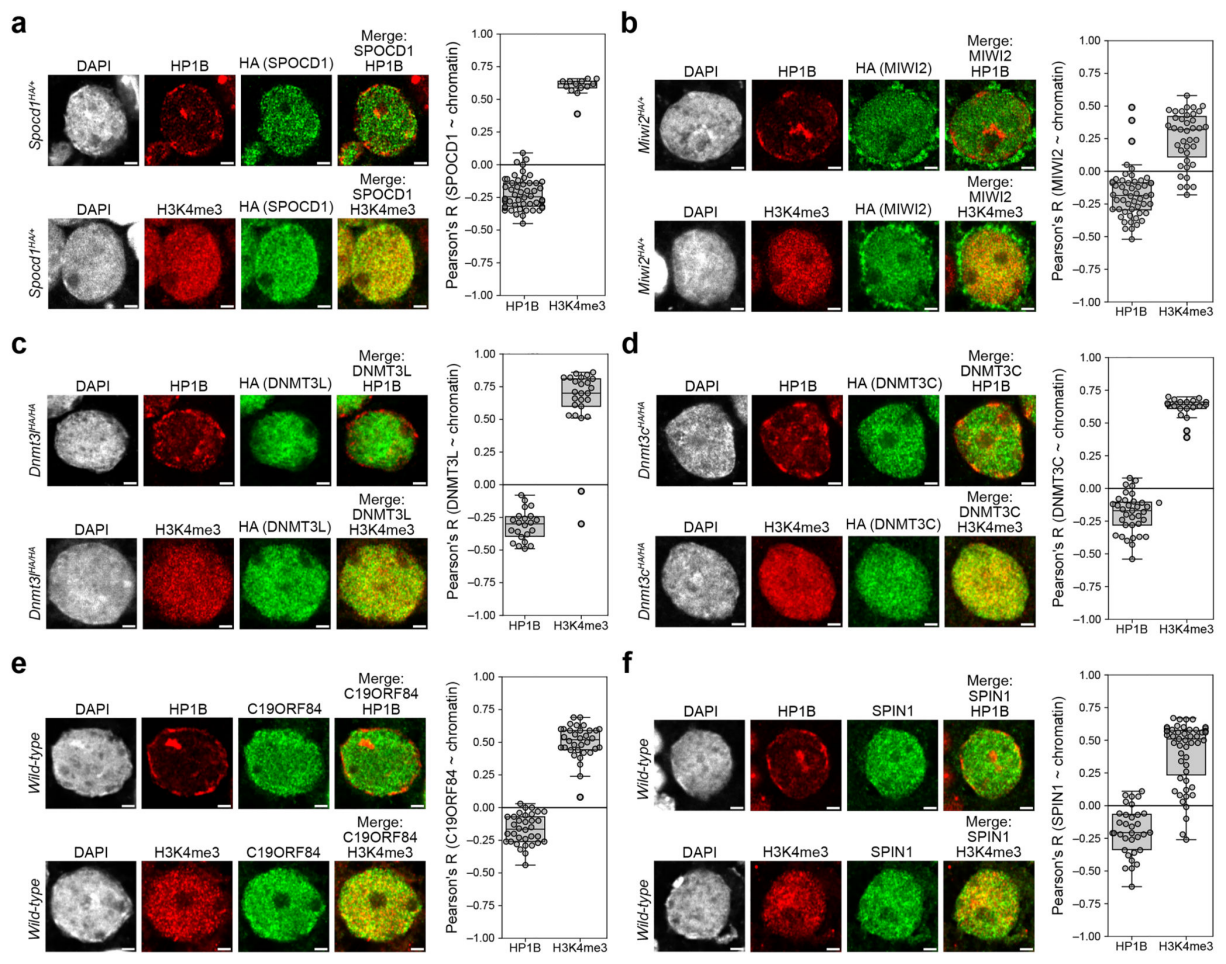
Nuclear piRNA factors and the *de novo* methylation machinery are euchromatic **a-d**, Representative E16.5 gonocyte stained for Haemagglutinin epitope tag (HA, green), HP1B (red, top row) or H3K4me3 (red, bottom row) and DAPI in foetal testis sections from *Spocd1*^*HA/+*^ (**a**), *Miwi2*^*HA/+*^ (**b**), *Dnmt3l*^*HA/HA*^ (**c**) and *Dnmt3c*^*HA/HA*^ (**d**) mice are shown. Shown to the right are boxplots of Pearson’s correlation coefficients of SPOCD1 (**a**), MIWI2 (**b**), DNMT3L (**c**) and DNMT3C (**d**) with HP1B and H3K4me3 in gonocyte nuclei. **e-f**, Representative E16.5 gonocyte stained for C19ORF84 (**e**) or SPIN1 (**f**) (both green), HP1B (red, top row) or H3K4me3 (red, bottom row) and DAPI in foetal testis sections from wild-type mice. Shown to the right are boxplots of Pearson’s correlation coefficients of C19ORF84 (**e**) and SPIN1 (**f**) with HP1B and H3K4me3 in gonocyte nuclei. Images in **a** through **f** are representative of n = 4 (**a, b, d**) or n = 3 (**c, e, f**) biological replicates; scale bars, 2 μm. Box indicates interquartile range, central line represents the mean, and whiskers extend to median ± 1.5× the interquartile range or data limits, whichever is smaller.

Source Data Figure 1

**Figure 2:**
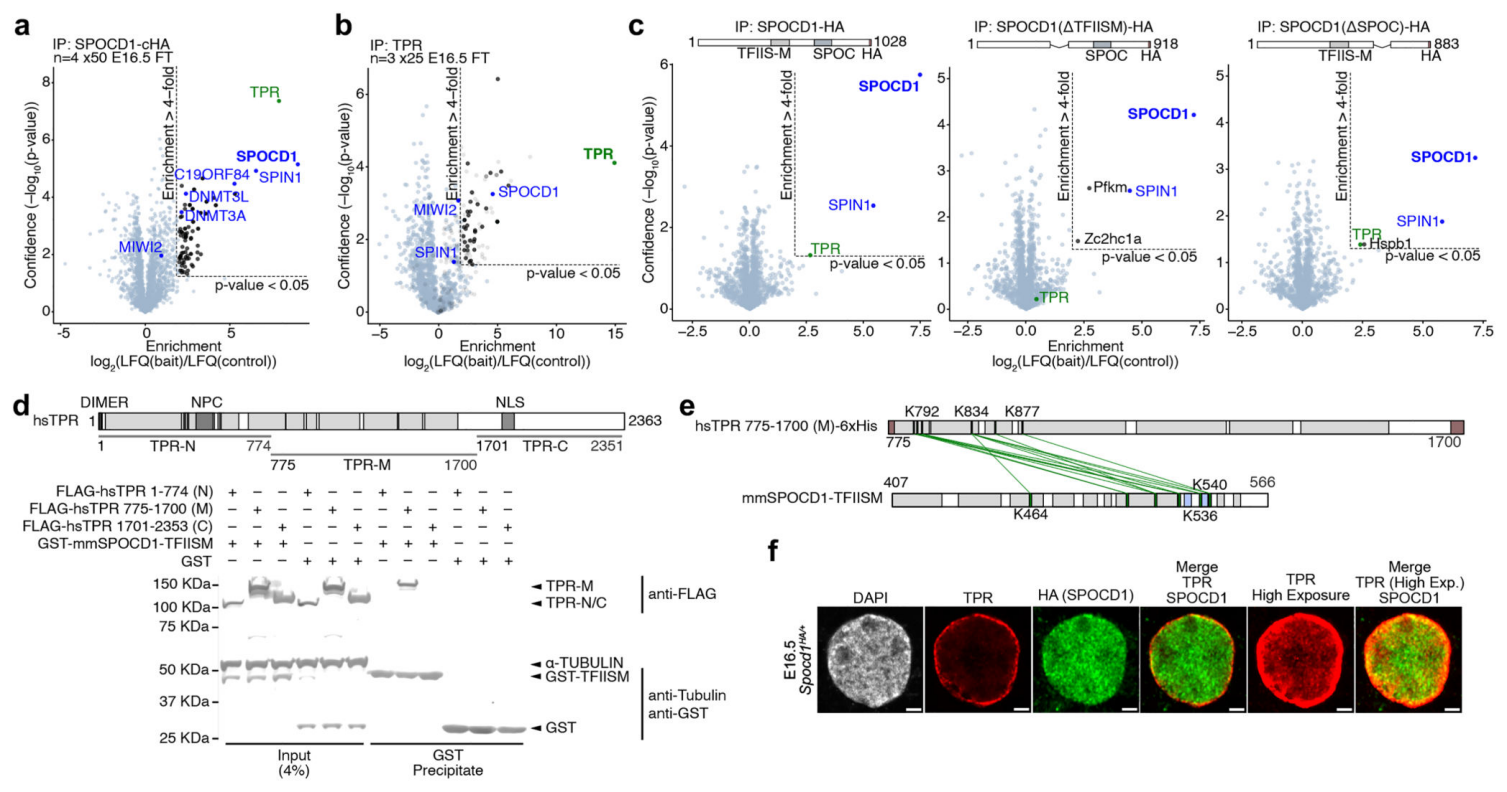
SPOCD1 directly interacts with TPR **a**, Volcano plot showing enrichment (log_2_(mean label-free quantification (LFQ) ratio of anti-HA immunoprecipitates from *Spocd1*^*HA/HA*^/wild-type E16.5 foetal testis lysates) and statistical confidence (−log_10_(P-value of two-sided Student’s t-test)) of proteins co-purifying with SPOCD1-HA (n = 4 with 50 testes per replicate, previously published data from ^19^). **b**, Volcano plot showing enrichment (log_2_(mean LFQ ratio of anti-TPR immunoprecipitated/anti-rabbit serum immunoprecipitated from wild-type E16.5 foetal testis lysates) and statistical confidence of proteins co-purifying with TPR (n = 3 with 25 foetal testes per replicate). All identified proteins meeting the enrichment cut-off are listed in Supplementary Table 1. Grey dots indicate IgG fragments. **c**, Volcano plots showing enrichment (log_2_(mean LFQ ratio of anti-HA immunoprecipitates from lysates of mouse embryonic stem cells (mESCs) ectopically expressing SPOCD1-cHA or the indicated domain deletion mutants/wild-type mESCs) and statistical confidence of proteins co-purifying with SPOCD1-HA (left), SPOCD1-ΔTFIISM-HA (centre) and SPOCD1-ΔSPOC-HA (right) (n = 3 independently derived SPOCD1-HA-expressing mESCs). **d**, Representative western blot image for n = 3 pull-down experiments with indicated mouse SPOCD1-TFIISM and lysates of HEK cells expressing the indicated human TPR fragments. For uncropped source gel image, see Supplementary Fig. 1a. **e**, Crosslinking mass spectrometry (CL-MS) of SPOCD1-TFIISM and recombinant human TPR-M. Inter-protein crosslinks and cross-linked lysine residues are shown in green. Grey regions indicate helical secondary structure, while blue regions indicate β-strands. More details about identified crosslinks are included in Supplementary Table 3. **f**, Representative E16.5 gonocyte stained for HA (SPOCD1-HA) (green), TPR (red) and DAPI in foetal testis sections from *Spocd1*^*HA/+*^ mice. Images are representative of n = 3 biological replicates. Scale bars, 2 μm.

Source Data Figure 2

**Figure 3:**
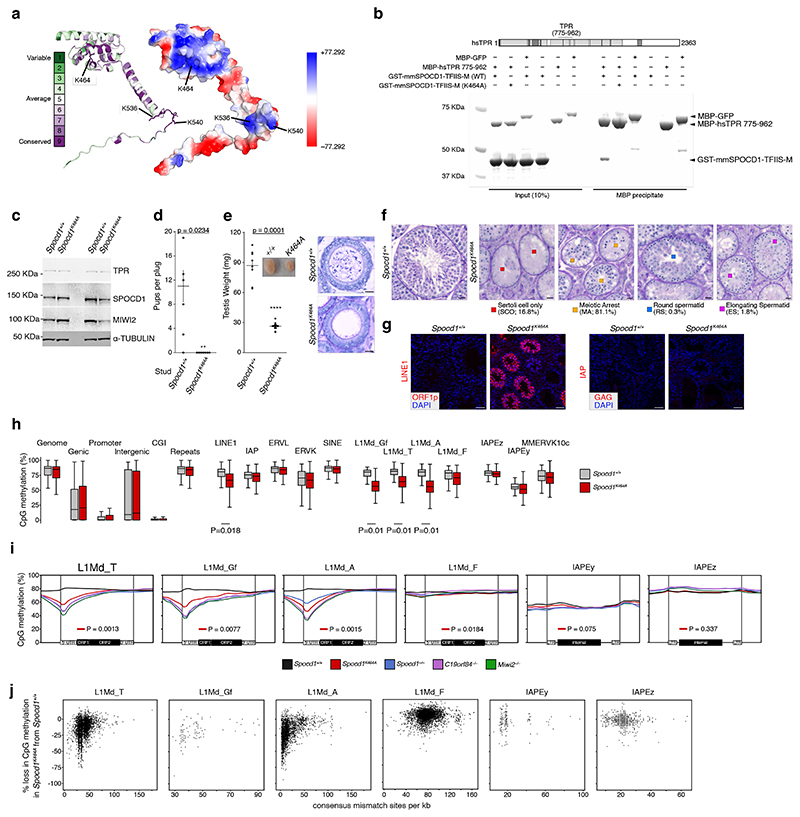
Defective *de novo* LINE1 methylation in *Spocd1*^*K464A*^ mice **a**, ConSurf conservation scores (left) and PyMOL predicted surface charge (right) of the mouse SPOCD1 TFIISM domain (amino acids 407-568, UniProt ID B1ASB6, AlphaFold 2 predicted structure). **b**, Representative Coomassie Blue-stained gel of n = 3 MBP pull-down experiments with the indicated recombinant mouse SPOCD1 and human TPR fragments. For uncropped source gel image, see Supplementary Fig. 1c. **c**, Representative western blot of n = 3 E16.5 foetal testis samples of the indicated genotypes indicating SPOCD1, MIWI2 and TPR protein levels in a single foetal testis. Alpha-tubulin served as loading control. For uncropped whole blot source images, see Supplementary Fig. 1b. **d**, Number of pups born per plug (mean ± SEM) fathered by n = 3 wild-type (6 plugs total) and n = 4 *Spocd1*^*K464A*^ studs (7 plugs total) mated to wild-type CD-1 females. **e**, Testis weight (mean ± SEM) of n = 8 wild-type and n = 16 *Spocd1*^*K464A*^ 12 week-old mice. Insert shows a representative image of testes from wild-type (left) and *Spocd1*^*K464A*^ (right) mice. Also shown are representative haematoxylin and periodic acid-Schiff (H&PAS)-stained epididymal tubules of n = 3 wild-type (top) and n = 4 *Spocd1*^*K464A*^ (bottom) mice. Scale bars, 20 μm. P-values in **d** and **e** are from unpaired two-tailed Student’s T-tests. **f**, Representative images of H&PAS-stained testes sections of wild-type and *n* = 3 *Spocd1*^*K464A*^ adult mice between 9 and 12 weeks of age, with different types of spermatogenic arrest observed in tubules of *Spocd1*^*K464A*^ testes. Scale bars, 20 μm. **g**, Representative adult testis sections of n = 3 wild type and *Spocd1*^*K464A*^ mice stained for DAPI (blue) and LINE1 ORF1p (red, left) or IAP GAG protein (red, right). Scale bars, 50 μm. **h-j**, Genomic CpG methylation analysis of P14 undifferentiated spermatogonia from wild-type (n = 4) and *Spocd1*^*K464A*^ (n = 3) mice. **h**, Percentages of CpG methylation levels of the indicated genomic features (with genic, promoter and CpG island (CGI) regions defined as those not overlapping transposable elements, and intergenic regions as those not overlapping transposable elements or genes) or transposable elements (not overlapping genes) are shown as box plots. Boxes represent interquartile range from 25^th^ to 75^th^ percentile, the horizontal line the median, whiskers the data range of the median ± 2× interquartile range. Significant differences (P-values < 0.05 of Benjamini-Hochberg-corrected unpaired two-tailed Student’s T-tests) to wild-type are indicated. **i**, Metaplots of mean CpG methylation over the consensus sequence of the indicated transposable elements and the adjacent 2 kb, comparing wild-type (n = 4), *Spocd1*^*K464A*^, *Spocd1*^*-/-*^, *C19orf84*^*-/-*^ and *Miwi2*^*-/-*^ P14 spermatogonia (all n = 3). P-values of Benjamini-Hochberg-corrected unpaired two-tailed Student’s T-tests comparing average CpG methylation of the promoter region (first 25 bins) to wild-type for *Spocd1*^*K464A*^. **j**, Mean CpG methylation loss relative to wild-type for individual transposons of the indicated families correlated with divergence from their consensus sequence in *Spocd1*^*K464A*^ spermatogonia.

Source Data Figure 3

**Figure 4:**
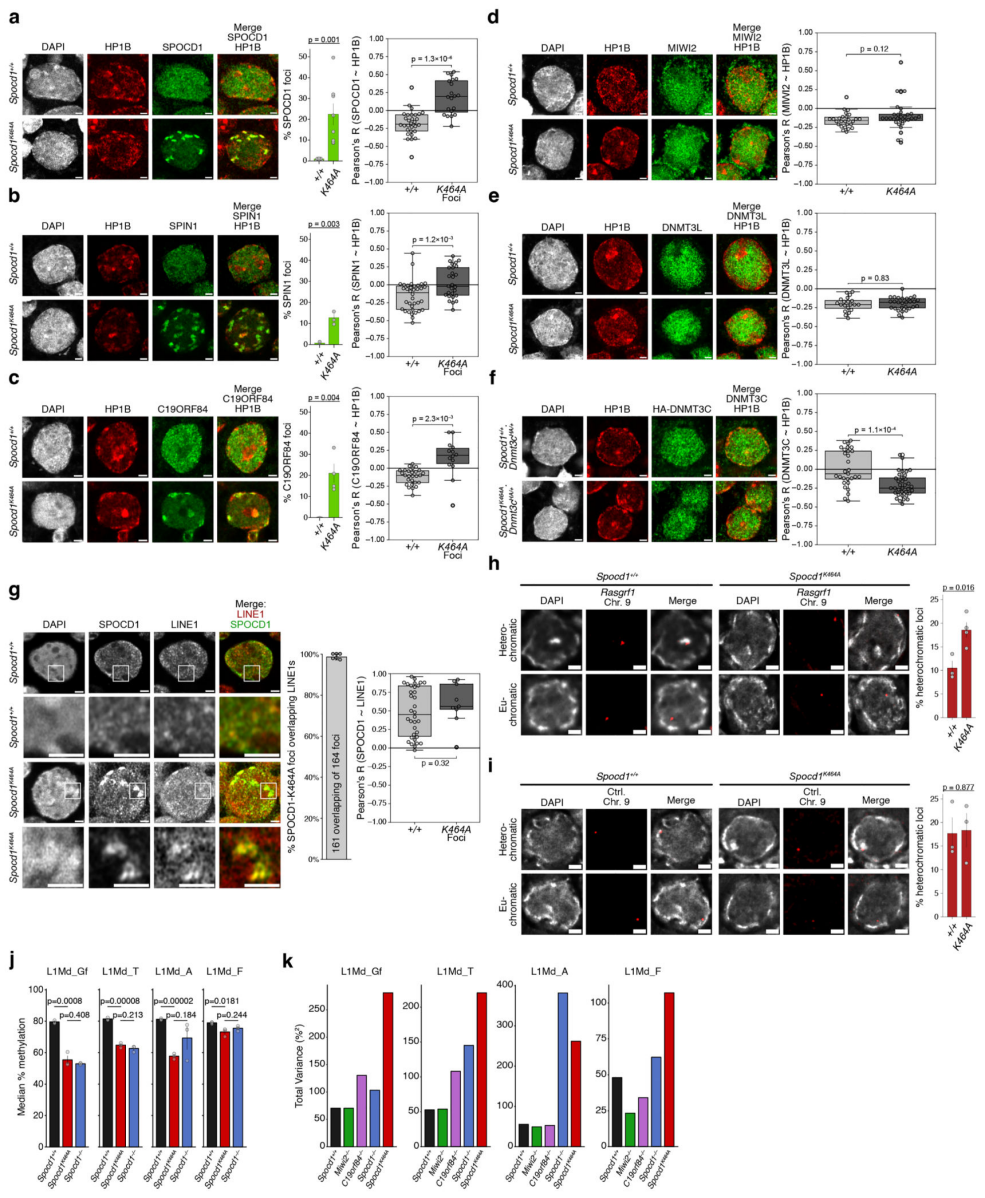
The SPOCD1-TPR interaction ensures LINE1 and *Rasgrf1* accessibility to MIWI2 and the *de novo* methylation machinery. **a-f**, Representative E16.5 gonocyte stained for DAPI, HP1B (red) and SPOCD1 (**a**), SPIN1 (**b**), C19ORF84 (**c**), MIWI2 (**d**), DNMT3L (**e**), or HA (HA-DNMT3C) (**f**) (all green) in wild-type (top row) or *Spocd1*^*K464A*^ (bottom row) foetal testis sections from littermates (n = 8 (**a**), n = 4 (**c**) or n = 3 (**b, d-f**) for each genotype). Shown to the right in **a-c** is the fraction of gonocytes with foci (mean ± SEM) for the stained proteins in sections from the indicated genotypes. Shown to the right in **a-f** are boxplots of Pearson’s correlation coefficients of the indicated proteins with HP1B in all wild-type and foci-containing *Spocd1*^*K464A*^ gonocyte nuclei (**a-c**), or all wild-type and *Spocd1*^*K464A*^ gonocyte nuclei (**d-f**). Scale bars, 2 μm. **g**, Representative E16.5 gonocyte (1^st^ and 3^rd^ rows) and zoomed-in view of region of interest (white rectangle on gonocyte nucleus) (2^nd^ and 4^th^ rows) stained for DAPI, SPOCD1 and LINE1 DNA in foetal testis sections of the indicated genotypes (n = 6 for each genotype). Shown to the right is the fraction (mean ± SD) of SPOCD1-K464A foci overlapping LINE1 DNA, as well as boxplots of Pearson’s correlation coefficients of SPOCD1 with LINE1 DNA in all wild-type and foci-containing *Spocd1*^*K464A*^ gonocyte nuclei. **a-g**, Box extends from 25^th^ to 75^th^ percentile, central line indicates the mean, whiskers extend between data limits or 1.5× the interquartile range, whichever is smaller. Scale bars, 2 μm. **h-i**, Representative E16.5 gonocyte with DAPI (greyscale) and DNA FISH for the *Rasgrf1* locus on Chr. 9 (**h**), or a control locus on Chr. 9 (**i**) (both red) in wild-type and *Spocd1*^*K464A*^ E16.5 foetal testis sections. An example of a heterochromatin- and an euchromatin- overlapping FISH signal in gonocytes of each genotype are shown as indicated (n = 3 for each genotype). Shown to the right is the fraction (mean ± SEM) of heterochromatin-overlapping signals in gonocytes in sections from the indicated genotypes. Scale bars, 2 μm. **j**, Median CpG methylation levels (%) (mean ± SEM) of the indicated LINE1 families in n = 3 biological replicates from the indicated genotypes. P-values are from unpaired two-tailed Student’s T-tests. **k**, Variance in CpG methylation levels (%^2^) of LINE1 loci across all replicates of the indicated genotypes (n = 3 for each genotype), summed over all loci within the indicated LINE1 families. P-values in **a-g** and **h-j** are from unpaired, two-tailed Student’s T-tests.

Source Data Figure 4

Supplementary Information

Supplementary Table 3

## Data Availability

The EM-seq data generated in this study have been deposited on ArrayExpress under accession number E-MTAB-14862. Data for the IP-MS experiments were deposited at ProteomeXchange under the accession number PXD060850. The CL-MS data is deposited under PXD060851. EM-seq data for *Spocd1*^*-/-*^ and *Miwi2*^*-/-*^ P14 spermatogonia were retrieved from E-MTAB-7997 (https://www.ebi.ac.uk/biostudies/arrayexpress/studies/E-MTAB-7997), while that for *C19orf84*^*-/-*^ spermatogonia were retrieved from E-MTAB-11612 (https://www.ebi.ac.uk/biostudies/arrayexpress/studies/E-MTAB-11612). The following Uniprot accession IDs for SPOCD1 proteins were used for conservation analyses: Mouse (*Mus musculus*, B1ASB6), Golden hamster (*Mesocricetus auratus*, A0A3Q0D6B7), Ord’s kangaroo rat (*Dipodomys ordii*, A0A1S3FIT4), Western European hedgehog (*Erinaceus europaeus*, A0A1S3WPZ3), Rabbit (*Oryctolagus cuniculus*, G1SPR0), Aardvark (*Orycteropus afer afer*, A0A8B7AXN8), Bison (*Bison bison bison*, A0A6P3HA20), Bovine (Bos taurus, F1MG39), Goat (*Capra hircus*, A0A452FMH8), Sheep (*Ovis aries*, W5NRM3), Pig (*Sus scrofa*, F1SV96), Horse (*Equus caballus*, F6YBJ1), Alpaca (*Vicugna pacos*, A0A6J3AYV9), California sealion (*Zalophus californianus*, A0A6J2C2W2), Northern fur seal (*Callorhinus ursinus*, A0A3Q7MZA7), Atlantic bottle-nosed dolphin (*Tursiops truncatus*, A0A6J3PXS9), Sperm whale (*Physeter macrocephalus*, A0A455B8T1), Blue whale (*Balaenoptera musculus*, A0A8B8W162), Great Himalayan leaf-nosed bat (*Hipposideros armiger*, A0A8B7SLV8), Large flying fox (*Pteropus vampyrus*, A0A6P3Q928), Leopard (*Panthera pardus*, A0A6P4UE11), Cat (*Felis catus*, A0A5F5XDK8), Red fox (*Vulpes vulpes*, A0A3Q7T0D7), Dog (*Canis lupus familiaris*, A0A8P0P5S7), Polar bear (*Ursus maritimus*, A0A8M1EZU5), Greater bamboo lemur (*Prolemur simus*, A0A8C8YEI0), Giant panda (*Ailuropoda melanoleuca*, G1MHH0), Rhesus macaque (*Macaca mulatta*, F7G2T4), Northern white-cheeked gibbon (*Nomascus leucogenys*, G1QNN7), Sumatran orangutan (*Pongo abelii*, H2N866), Gorilla (*Gorilla gorilla gorilla*, G3RKR7), Chimpanzee (*Pan troglodytes*, H2R1B9), Human (*Homo spaiens*, Q6ZMY3), American alligator (*Alligator mississippiensis*, A0A151MMW3), Chinese alligator (*Alligator sinensis*, A0A1U8CWC7), Snapping turtle (*Chelydra serpentina*, A0A8T1SPX3), Central bearded dragon (*Pogona vitticeps*, A0A6J0UYI0), Mainland tiger snake (*Notechis scutatus*, A0A6J1UEZ9), Indian cobra (*Naja naja*, A0A8C6YBU2), American chameleon (*Anolis carolinensis*, H9GI50), Western clawed frog (*Xenopus tropicalis*, A0A8J0T0T7), African clawed frog (*Xenopus laevis*, A0A1L8HFK1).

## Code availability

Scripts used for the EM-seq analysis are available on GitHub (https://github.com/tamchow/spocd1_pirna-directed-dna-met-variance/ (version of record deposited at Zenodo, DOI: 10.5281/zenodo.17162836) and https://github.com/rberrens/SPOCD1-piRNA_directed_DNA_met (version of record deposited at Zenodo, DOI: 10.5281/zenodo.10509247)). The Fiji/ImageJ2 plugin used to calculate Pearson’s correlation coefficients in germ cell nuclei is also available on Github (https://github.com/COIL-Edinburgh/ROI_NucleusColocalisation; a version of record has been deposited at Zenodo with DOI: 10.5281/zenodo.17200734).
