# Supplementary material for: A nowhere-to-hide mechanism ensures complete piRNA-directed DNA methylation
Source: Nature. Author manuscript; Available in PMC 2026 Feb 19. (PMC7618654; doi:10.1038/s41586-025-09940-w)
Supplement: Supplementary Information [file EMS212078-supplement-Supplementary_Information.pdf]

A nowhere-to-hide mechanism ensures complete piRNA-directed DNA methylation

Tamoghna Chowdhury [1,2], Shelagh Boyle[3], Ansgar Zoch[1,2,3], Xinyu Xiang[1,2,4], Madeleine Dias Mirandela[1,2], Hanna Fieler[1,2], Christos Spanos[2], Juan Zou[2], David Kelly[2], Wendy A. Bickmore[3], Atlanta G. Cook[2] and Dónal O'Carroll[1,2,*]

[1]Centre for Regenerative Medicine, Institute for Regeneration and Repair, Institute for Stem Cell Research, University of Edinburgh, 5 Little France Drive, Edinburgh, EH16 4UU, UK.

[2]Centre for Cell Biology, University of Edinburgh, Michael Swann Building, Max Born Crescent, Edinburgh, EH9 3BF, UK.

[3]MRC Human Genetics Unit, Institute of Genetics and Cancer, University of Edinburgh, Crewe Road South, Edinburgh EH4 2XU, Edinburgh, UK

[4]Zhejiang University-University of Edinburgh Institute (ZJU-UoE Institute), Zhejiang University School of Medicine, International Campus, Zhejiang University, 718 East Haizhou Road, Haining 314400, China

Supplementary Information Table of Contents

Supplementary Figures:

Figure	Figure Title	Page
1	Un-cropped Western Blot and Coomassie-stained SDS-PAGE gels	3
2	FACS gating strategies	4

Supplementary Tables:

Table	Table Title	Page
1	Proteins identified as TPR interactors in E16.5 foetal testes	5
2	Proteins gaining or losing association with SPOCD1-K464A in E16.5 foetal testes	6
3	Identified cross-links between SPOCD1-TFIISM and TPR-M	6

Supplementary Information summary

Supplementary information containing uncropped scans of Western blot experiments shown in Figures 2d and 3c, as well as uncropped scans of Coomassie blue stained SDS-PAGE gels shown in Figure 3b and Extended Data Figure 4b (Supplementary Figure 1), the FACS gating strategy for sorting undifferentiated spermatogonia and GFP^+ mESCs (Supplementary Figure 2) and three additional data tables (Supplementary Tables 1-3) relating to IP-MS data (Supplementary Tables 1-2) and CL-MS data (Supplementary Table 3).

Supplementary Figures

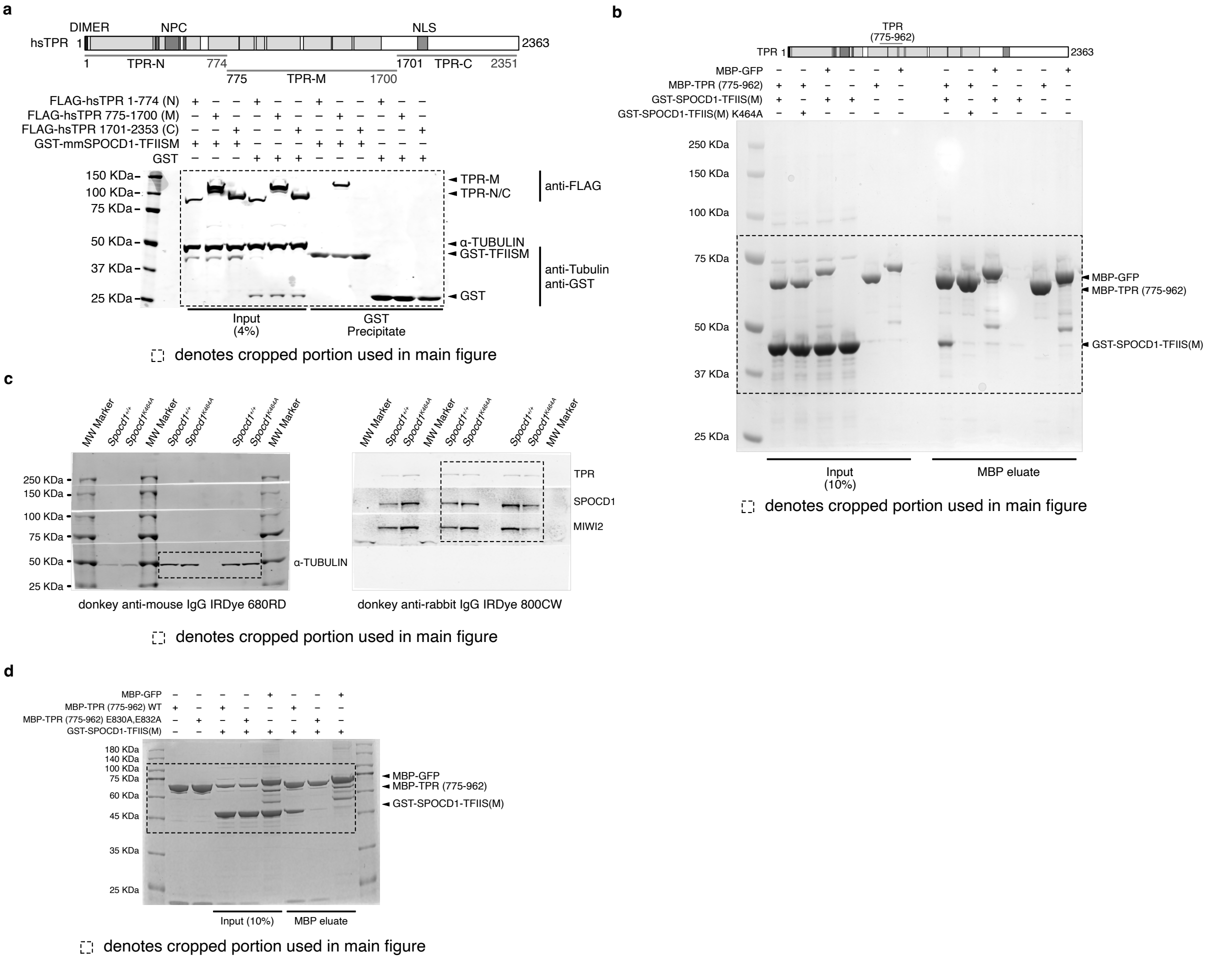

Supplementary Figure 1. Un-cropped Western Blot and Coomassie-stained SDS-PAGE gels.

- **a.** Un-cropped Western blot image representative of n = 3 pull-down experiments with the indicated recombinant mouse SPOCD1 fragments and HEK293T cell lysates expressing human TPR fragments, relevant to main text Fig. 2d. Alpha-tubulin served as loading control.
- **b.** Un-cropped Coomassie blue stained SDS-PAGE gel image representative of n = 3 MBP pull-down experiments with the indicated recombinant mouse SPOCD1 and human TPR fragments, relevant to main text Fig. 3b.
- **c.** Un-cropped Western blot image representative of n = 2 independent experiments showing TPR, SPOCD1 and MIWI2 levels in lysates of single foetal testis of n = 3 different embryos of the indicated genotypes per experiment, relevant to main text Fig. 3c. Alpha-tubulin served as loading control.

d. Un-cropped Coomassie blue stained SDS-PAGE gel image representative of n = 3 MBP pull-down experiments with the indicated recombinant mouse SPOCD1 and human TPR fragments, relevant to Extended Data Fig. 4b.

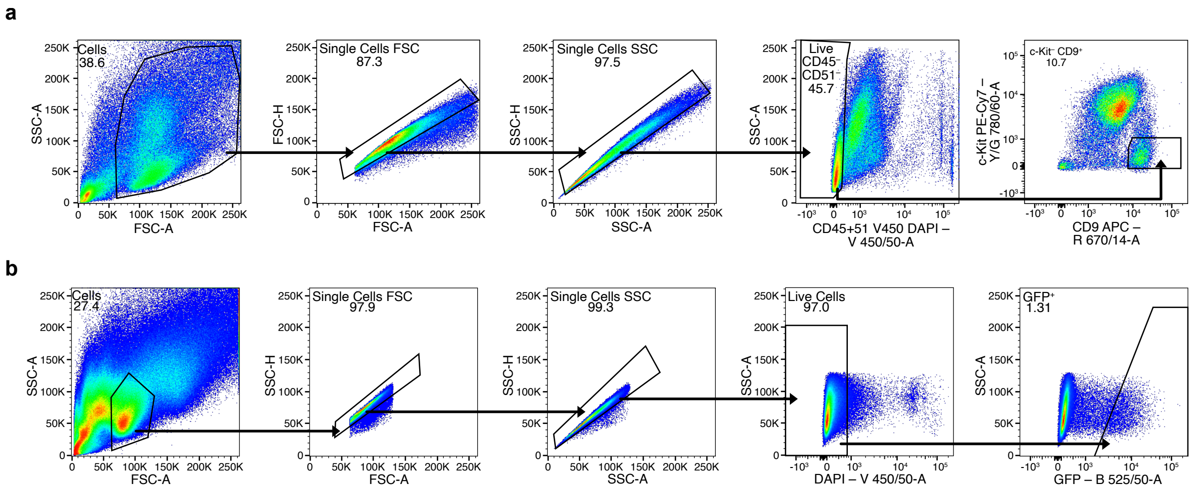

Supplementary Figure 2. FACS gating strategies

a. FACS gating strategy to obtain $CD45^{-}$ $CD51^{-}$ $c\text{-}kit^{-}$ $CD9^{+}$ undifferentiated spermatogonia from single cell suspension of P14 juvenile mouse testes. Representative of n = 8 sorting experiments total.

b. FACS gating strategy to obtain GFP^{+} mouse embryonic stem cells (mESCs) after transfection with piggybac plasmids encoding CAG promoter-SPOCD1-HA-IRES-EGFP cassettes. Cells were sorted twice within a 1-week interval using the same gating strategy to confirm stable expression of cassette. Representative of n = 24 sorting experiments total.

Supplementary Tables

Uniprot ID	Gene name	p-value	Fold change
Q7M739	Tpr	7.4978E-05	14.9666138
P62305	Snrpe	0.00031773	5.96588326
P10404	env	0.00013037	5.34221776
Q9R1A8	Rfwd2	3.6734E-07	5.0571537
Q8BFY9	Tnpo1	0.00313708	5.01634916
P26151	Fcgr1	0.00016231	4.94757907
B1ASB6	Spocd1	0.00054306	4.6226902
Q9D5K4	S100pbp	0.0001418	4.43505542
F6ZDS4	Tpr	0.00017673	4.06725121
Q80YV2	Zc3hc1	0.00135035	4.06634458
Q9ESW4	Agk	0.00430585	3.72880491
Q9Z2N8	Actl6a	0.00103982	3.33991178
Q9ESZ8	Gtf2i	0.00596027	3.12375323
P58468	Fam207a	0.00542767	3.12336731
Q9ERF3	Wdr61	0.00953785	3.07462057
P23116	Eif3a	0.01862118	3.05967776
P07724	Alb	0.01100979	3.02528763
Q8CAB7	Fam98b	0.04728941	2.89936829
Q05512	Mark2	0.00048976	2.85830053
Q3U1J4	Ddb1	0.03741147	2.76811854
Q9Z315	Sart1	0.00088538	2.7388045
Q6P4T2	Snrnp200	0.00165161	2.72204399
Q9QZQ8	H2afy	0.01039749	2.71286202
Q9DBG6	Rpn2	0.04011196	2.67426618
Q9D6Z1	Nop56	0.00209193	2.57036972
Q9WTX8	Mad1l1	7.8102E-05	2.50903066
Q9Z1N5	Ddx39b	0.01919403	2.49186389
Q8C0C7	Farsa	0.00165923	2.49150848
P47753	Capza1	0.01486898	2.48596191
Q8BGX1	Pced1b	0.00398657	2.47314453
Q6NZN0	Rbm26	0.00492272	2.33296331
P70388	Rad50	0.03196368	2.28264109
Q9Z110	Aldh18a1	0.01146322	2.26482646
Q8CDG1	Piwil2	0.00282531	2.23621241
Q8C804	Spice1	0.01905745	2.2209657
Q810V0	Mphosph10	0.00442225	2.19789759
Q8CDP6	Skiv2l	0.00783813	2.13819567

Supplementary Table 1. Proteins identified as TPR interactors in E16.5 foetal testes.

Table listing all statistically significant (P < 0.05, two-sided Student's t-test, n=3) proteins that are at least 4-fold enriched in the TPR immuno-precipitation (IP) over the rabbit serum IP, relevant to Fig. 2b.

**Uniprot ID**	**Gene name**	**p-value SPOCD1-WT vs. Spocd1-K464A/K464A**	**Fold change SPOCD1-WT vs. Spocd1-K464A/K464A**
Q7M739	Tpr	0.01021349	4.818681717
Q8K370	Acad10	0.019208155	2.688751221
Q9CPP6	Ndufa5	0.040907792	2.515228907
P14733	Lmnb1	0.033145538	-2.004037221
B1ASB6	Spocd1	0.040996815	-2.014989217
P20152	Vim	0.003356983	-2.062614441
Q61164	Ctcf	0.049585591	-2.437076569
Q9Z2U1	Psma5	0.025081315	-2.625517527
Q61686	Cbx5	0.021946828	-2.784540812
P02772	Afp	0.02501074	-2.951210658
P02468	Lamc1	0.01616317	-3.321629842
E9PZ16	Hspg2	0.044416211	-3.622854869
Q61879	Myh10	0.029432604	-3.893040339
P21981	Tgm2	0.004889922	-4.449165344
P11276	Fn1	0.043785523	-4.529900869

Supplementary Table 2. Proteins gaining or losing association with SPOCD1-K464A in E16.5 foetal testes.

Table listing all statistically significant (P < 0.05, two-sided Student's t-test, n=3) proteins that are at least 4-fold enriched in either the SPOCD1 IP from wild-type or from *Spocd1*^{K464A} E16.5 foetal testes, relevant to Extended Data Fig. 5c.

(Attached Excel file: Supplementary Table 3.xlsx)

Supplementary Table 3. Identified cross-links between SPOCD1-TFIISM and TPR-M.

Table listing all Xi-validated cross-linked peptides detected in and between SPOCD1-TFIISM and TPR-M for n=2 cross-linking reactions (analysed together), relevant to Fig. 2e.
